# CT-Based Radiomics in the Characterization of Solid Renal Tumors: A Systematic Review

**DOI:** 10.3390/cancers18111758

**Published:** 2026-05-27

**Authors:** Petros Koumpis, Eyrysthenis Vartholomatos, Eleni Romeo, George A. Alexiou, Maria I. Argyropoulou, Athina C. Tsili

**Affiliations:** 1 Department of Clinical Radiology, University Hospital of Ioannina, University Campus, 45110 Ioannina, Greece; petroskoumpis@gmail.com (P.K.); eyrys.varth@gmail.com (E.V.); 2 Department of Neurosurgery, Faculty of Medicine, School of Health Sciences, University of Ioannina, University Campus, 45110 Ioannina, Greece; e.romeo@uoi.gr (E.R.); galexiou@uoi.gr (G.A.A.); 3 Department of Clinical Radiology, Faculty of Medicine, School of Health Sciences, University of Ioannina, University Campus, 45110 Ioannina, Greece; margyrop@uoi.gr

**Keywords:** artificial intelligence, machine learning, computed tomography, radiomics, renal cell carcinoma, renal neoplasms

## Abstract

**Background:** Renal cell carcinoma (RCC) is a global health challenge characterized by significant histological heterogeneity. Conventional contrast-enhanced CT often struggles to differentiate RCC from solid benign renal tumors like fat-poor angiomyolipoma (fpAML) and renal oncocytoma (RO), leading to potential surgical overtreatment. CT-based radiomics has emerged as a promising non-invasive approach that extracts high-dimensional quantitative imaging features to support lesion characterization and may contribute toward more comprehensive, biopsy-adjacent decision support, although it does not yet replace histopathological assessment. **Methods:** This review systematically evaluates the predictive performance of CT-based radiomics in characterizing solid renal tumors. A literature search was conducted in PubMed/MEDLINE, Cochrane, and Scopus databases for original research published between 2012 and 2025. The review focuses on four key areas: differentiating benign renal tumors from RCC, clear cell (ccRCC) from non-ccRCC, fpAML from RCC, and RO from RCC. **Results:** In total, 47 studies were assessed, including 11,999 patients. CT-based radiomics demonstrates high diagnostic performance across all categories. Median Area Under the Curve values were 0.830 (0.747–0.900) for benign vs. malignant differentiation, 0.900 (0.861–0.910) for ccRCC vs. non-ccRCC, 0.912 (0.879–0.933) for fpAML vs. RCC, and 0.885 (0.841–0.947) for RO vs. RCC. The integration of radiomic features with clinical parameters into combined nomograms consistently yielded the highest predictive accuracy. **Conclusions:** Radiomics provides a non-invasive, objective method to characterize renal tumors, potentially reducing unnecessary surgeries and enabling personalized treatment. However, widespread clinical adoption remains limited by a lack of protocol standardization, the need for automated segmentation, and the requirement for prospective, multicenter validation.

## Simple Summary

Renal cell carcinoma (RCC) presents a significant diagnostic challenge due to its marked heterogeneity and the high prevalence of benign tumors, such as fat-poor angiomyolipoma (fpAML) and renal oncocytoma (RO), which are frequently overtreated surgically. This systematic review, encompassing 47 studies and 11,999 patients, evaluates the efficacy of CT-based radiomics as a “virtual biopsy” tool for the non-invasive characterization of solid renal tumors. The analysis demonstrates high diagnostic accuracy, with a median Area Under the Curve of 0.830 (0.747–0.900) for differentiating benign tumors from RCC, 0.900 (0.861–0.910) for clear cell RCC vs. non-clear cell RCC discrimination, 0.912 (0.879–0.933) for fpAML vs. RCC identification, and 0.885 (0.841–0.947) for RO vs. RCC differentiation. Notably, combined nomograms provide the most accurate predictions, although the number of qualifying studies remains small. Ultimately, CT radiomics offers a non-invasive method for repeated evaluation of intratumoral heterogeneity, with potential applications in personalized treatment strategies.

## 1. Introduction

Renal cell carcinoma (RCC) has emerged as a significant global health concern, representing approximately 2% to 3.5% of all adult malignancies. While it ranks 14th among cancers worldwide, it is the most common solid tumor of the kidney, with the highest incidence rates observed in Western countries [1]. Over the past few decades, the widespread expansion of cross-sectional imaging—such as ultrasound and CT—has driven a steady increase in detected cases; as roughly 60% to 67% of RCC cases are diagnosed incidentally, a shift that has led to a notable trend toward the detection of smaller, lower-stage asymptomatic tumors [1,2,3,4].

RCC is not a single disease but a heterogeneous group of tumors with vastly different biological behaviors. There are three main RCC types: clear cell (ccRCC), papillary (pRCC) and chromophobe RCC (chRCC) [5,6]. The pretreatment identification of major histologic subtypes of RCC is important because it directly dictates the clinical management, therapeutic approach, and prognostic expectations for a patient [1,2,3,4].

In addition, the clinical challenge of managing solid renal masses is underscored by the high prevalence of benign pathology, which accounts for approximately 20% of all surgically resected solid renal tumors. Among these, fat-poor angiomyolipoma (fpAML) and renal oncocytoma (RO) represent the most frequent benign diagnoses [2,3,7,8]. Historically, many of these lesions were treated with radical or partial nephrectomy (PN); however, such “overtreatment” incurs unnecessary healthcare costs and exposes patients to avoidable surgical risks and loss of renal function. Consequently, the development of accurate, noninvasive characterization techniques is essential for better tailoring therapeutic planning [2,3,7,8,9,10].

Radiologists have a key role in the diagnosis, characterization, and staging of RCC [1,2,3,4,5,6,7,8,9,10,11,12]. Contrast-enhanced CT (CECT) is often used for the characterization of solid renal tumors and is widely accepted as the diagnostic modality of choice for RCC staging. However, the technique cannot always characterize solid renal tumors based on qualitative features [3,7,8,9,10].

Radiomics represents a transformative advancement in medical imaging, shifting from conventional visual interpretation to the high-throughput extraction of quantitative data. First conceptualized by Lambin et al. in 2017 to address solid tumor heterogeneity, radiomics utilizes advanced computational analysis to convert radiological images into mineable, high-dimensional datasets [13]. By employing complex image processing technologies—including texture analysis and machine learning (ML) algorithms—this approach captures subtle features like shape, intensity, and heterogeneity within Volumes of Interest (VOIs) that are often imperceptible to the human eye. These quantitative features allow for the creation of descriptive phenotypes and predictive models by integrating imaging data with genomic and protein signatures [13,14,15,16,17].

Functioning as a “virtual biopsy,” radiomics offers a non-invasive, objective method to assess both intra-tumoral and inter-tumoral heterogeneity, with the distinct advantage of repeatability over time. In clinical settings, particularly for renal masses and RCC, CT-based radiomics has demonstrated significant utility in tumor characterization, RCC grading, prognostication, and the assessment of therapeutic response. By augmenting traditional diagnostics with these comprehensive, microscale insights, radiomics enhances diagnostic accuracy and supports personalized medicine, ultimately aiding clinicians in improving decision-making and patient outcomes in oncological care [18,19,20,21,22,23,24,25,26,27,28,29,30].

A systematic review of the literature, including 13 studies, reported that CT radiomic pipelines have robust performance in differentiating between renal tumor types, achieving an Area Under the Curve (AUC) of 0.915 [28].

The present systematic review aims to comprehensively update the role of CT-based radiomics in the characterization of solid renal tumors. It specifically focuses on the differentiation of benign from malignant renal tumors, clear cell RCC from non-clear cell RCC, fat-poor angiomyolipoma from RCC, and renal oncocytoma from RCC.

## 2. Methods

This systematic review strictly follows the Preferred Reporting Items for Systematic Reviews and Meta-Analyses (PRISMA) guidelines [31]. The protocol for this study was registered in PROSPERO under the registration number: CRD420261302280.

### 2.1. Literature Search Strategy and Selection Criteria

To conduct this systematic review, a comprehensive literature search was performed within the PubMed/MEDLINE, Cochrane and Scopus databases, spanning from 2012 through 30 November 2025.

The search strategy utilized key terms including “renal” OR “kidney”, “renal tumors” OR “renal masses”, “Renal Cell Carcinoma” OR “RCC”, “computed tomography” OR “CT”, “radiomics” OR “artificial intelligence”, and “machine learning” OR “ML”. Following the removal of duplicate records, three independent investigators (PK, EV and ER) screened titles and abstracts to exclude irrelevant studies. The full texts of the remaining studies were then obtained and reviewed according to predefined inclusion and exclusion criteria. Any discrepancies during the screening process were resolved via consultation with the corresponding author.

The inclusion criteria were as follows: studies conducted on patients with solid renal tumors, characterized by surgical histopathology (total nephrectomy, PN or core biopsy); radiomics features extraction from renal tumors based on CT; availability of information for extraction of pipeline characteristics, such as CT acquisition parameters, segmentation methods, radiomics features used, ML models, and classification results compared with histology; validation performed on an independent cohort separate from the training cohort; and reported AUC for renal tumor characterization.

**Figure 1:**
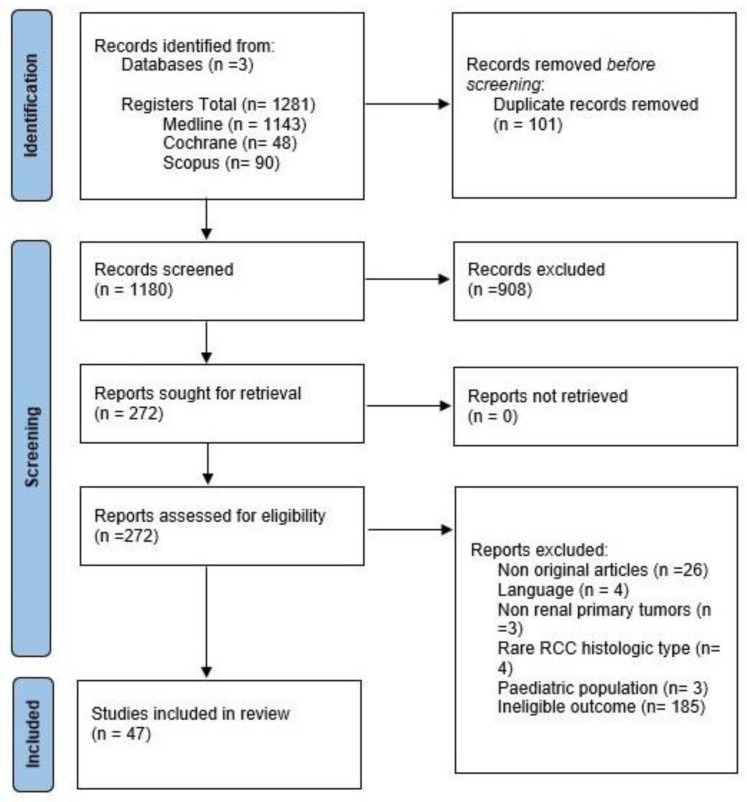
Flowchart depicting study selection.

Studies were excluded based on the following criteria: (1) non-original research, including reviews, editorials, case reports/series, conference abstracts, and book chapters; (2) non-English-language publications; (3) non-solid, non-primary renal tumors or rare RCC histological subtypes; (4) focus on pediatric populations; and (5) irrelevant clinical outcomes. This rigorous selection process, supplemented by a manual screening of reference lists, culminated in the final cohort of studies as detailed in the flow chart in Figure 1.

### 2.2. Data Extraction

From each original study, the following design characteristics were recorded: first author and year of publication; study design (prospective or retrospective; single-center or multicenter); primary outcome; and characteristics of the study population, including the number of patients with renal tumors, number and histology of renal tumors, age, sex, tumor size, and type of surgery.

CT imaging characteristics included detailed information on the following: type of CT scanner; slice thickness, kilovolt (kV) and milliampere (mA) used, type and amount of intravenous (iv) contrast medium, rate of injection, phases and timing of CECT phases obtained.

Radiomics process characteristics detailed the following: segmentation method, radiomics features (RFs) extracted, feature selection techniques, and algorithms used to construct the model. The AUC was recorded as the primary indicator of the predictive performance of the radiomics signature in the validation cohorts. When multiple validation cohorts were reported, data from the external validation cohort were preferentially extracted, prioritizing the cohort with the largest sample size. In the absence of external validation, results from the internal validation cohort with the largest sample size were selected.

When at least two datasets were available, we extracted data from clinical models (including clinical information and/or morphological CT features), and radiomics nomograms. Data extraction was conducted independently by three researchers (PK, EV, and ER), and any discrepancies were discussed with the corresponding author.

Due to the absence of sufficient and appropriate quantitative data, as well as substantial clinical and methodological heterogeneity among the included studies, a meta-analysis was not feasible. Instead, this review provides a structured overview of the role of CT-based radiomics in characterizing solid renal tumors across the following subcategories: benign versus malignant; ccRCC versus non-ccRCC; fpAML versus RCC; and RO versus RCC.

### 2.3. Quality Assessment

The methodological quality and potential bias of the included studies were assessed using QUADAS-2, and the results were presented graphically [32]. Two reviewers (ER and PK) conducted the assessment independently.

The QUADAS-2 tool evaluates potential sources of bias across four domains: patient selection, index test, reference standard, and flow and timing. It is also used to assess applicability concerns across the patient selection, index test, and reference standard domains. Each domain is judged as having a low, high, or unclear risk of bias based on responses to predefined signaling questions [32].

Methodological quality was also evaluated using the METhodological RadiomICs Score (METRICS), which includes 30 weighted items across multiple domains. Each item is rated “yes” or “no,” producing a percentage score that classifies studies as very low, low, moderate, good, or excellent quality [33].

Discrepancies between reviewers were resolved by the corresponding author.

### 2.4. Statistical Analysis

Statistical analyses were performed using R version 4.5.2 (R Foundation for Statistical Computing, Vienna, Austria) and RStudio version 2026.01.0 (Posit Software, PBC, Boston, MA, USA). Due to the absence of uniformly reported summary statistics across the included studies, aggregate estimates for mean age and tumor size, along with their corresponding standard deviations (SDs), were calculated using weighted methods. Weighted means were derived from study-specific means and sample sizes, assigning greater weight to studies with larger populations. Pooled standard deviations were calculated by combining within-study and between-study variability using standard statistical formulas.

A descriptive statistical analysis was conducted to evaluate model performance using the AUC. For studies reporting multiple models for the same diagnostic outcome, a single study-level AUC was calculated by averaging the reported validation AUCs before descriptive aggregation across studies. This approach was adopted to reduce the disproportionate influence of studies reporting multiple models and to avoid treating multiple models from the same study as independent observations. External or internal validation cohort results were prioritized over training data.

For each model category and clinical outcome, summary statistics of AUC values were calculated, including mean, standard deviation (SD), median, interquartile range (IQR), and range. Only AUC point estimates were included in the pooled descriptive analysis, as measures of variability (e.g., SDs or confidence intervals) were inconsistently reported across studies.

A separate within-study comparative analysis was performed for studies that evaluated multiple models within the same study population. This approach allowed direct comparison of model performance while minimizing bias related to differences in patient populations and study design. Differences in AUC values between models were calculated and summarized descriptively.

## 3. Results

### 3.1. Search Finding

An initial search across PubMed/MEDLINE (*n* = 1143), Cochrane (*n* = 48), and Scopus (*n* = 90) yielded a total of 1281 records. After removing 101 duplicates, 1180 articles underwent title and abstract screening. Preliminary screening and reference cross-checking identified 272 potentially relevant studies, of which 47 met the final inclusion criteria for analysis (Figure 1) [34,35,36,37,38,39,40,41,42,43,44,45,46,47,48,49,50,51,52,53,54,55,56,57,58,59,60,61,62,63,64,65,66,67,68,69,70,71,72,73,74,75,76,77,78,79,80].

### 3.2. Risk of Bias and Applicability Assessment

A total of 47 studies [34,35,36,37,38,39,40,41,42,43,44,45,46,47,48,49,50,51,52,53,54,55,56,57,58,59,60,61,62,63,64,65,66,67,68,69,70,71,72,73,74,75,76,77,78,79,80] were assessed for risk of bias across the domains of patient selection, index test, reference standard, and flow and timing domains using the QUADAS-2 tool [32], revealing concerns regarding selection bias, while demonstrating high institutional adherence to reference standards. Specifically, in the patient selection domain, 41 studies were rated high-risk [35,36,37,38,39,40,41,42,43,44,45,46,47,48,49,50,51,52,53,54,55,56,57,58,59,60,61,62,63,64,65,66,67,68,69,70,71,73,74,76,77,80], four low-risk [34,66,72,78], and two unclear [75,79]. For the index test domain, most studies were unclear (*n* = 25) [39,40,42,45,46,47,50,53,54,56,57,58,59,60,62,63,64,68,69,71,72,73,76,79,80], with 19 low-risk [34,35,36,38,41,44,48,49,51,55,61,65,66,67,70,74,75,77,78], and three high-risk [37,43,52]. In the reference standard domain, 46 studies were low-risk [34,35,36,37,38,39,40,41,42,43,44,45,46,47,48,49,50,51,52,53,54,55,56,57,58,59,60,61,62,63,64,65,66,67,68,70,71,72,73,74,75,76,77,78,79,80], and one high-risk [69]. In the flow and timing domain, 26 studies were low-risk [35,38,42,44,45,46,48,49,51,52,58,60,63,65,66,67,71,72,74,75,76,77,79], 19 unclear [36,37,39,47,48,49,50,51,52,53,61,62,64,68,69,70,73,78], and two high-risk [34,43].

QUADAS-2 was used to assess applicability concerns across the patient selection, index test, and reference standard domains, revealing moderate concerns regarding the applicability of patient cohorts. Specifically, for patient selection, 28 studies showed high concerns [34,35,36,37,39,40,41,42,43,45,46,47,48,54,56,57,59,63,64,65,66,67,68,69,71,73,74,76,80], 15 low concerns [38,39,49,50,51,53,55,58,60,61,62,70,72,77,78] and four unclear [44,52,75,79]. In the index test domain, 29 studies had low concerns [34,35,36,37,38,39,40,41,42,44,45,46,47,48,49,51,52,55,61,64,65,66,67,68,70,74,75,77,78], and 18 were unclear [43,50,53,54,56,57,58,59,60,62,63,69,71,72,73,76,79,80]. In the reference standard domain, 44 studies had low concerns [34,35,36,37,38,39,40,41,42,43,44,45,46,47,48,49,50,51,52,53,54,55,56,57,58,59,60,61,62,63,65,66,67,70,71,72,73,74,75,76,77,78,79,80], two were unclear [64,68], and one had high concerns [69].

**Figure 2:**
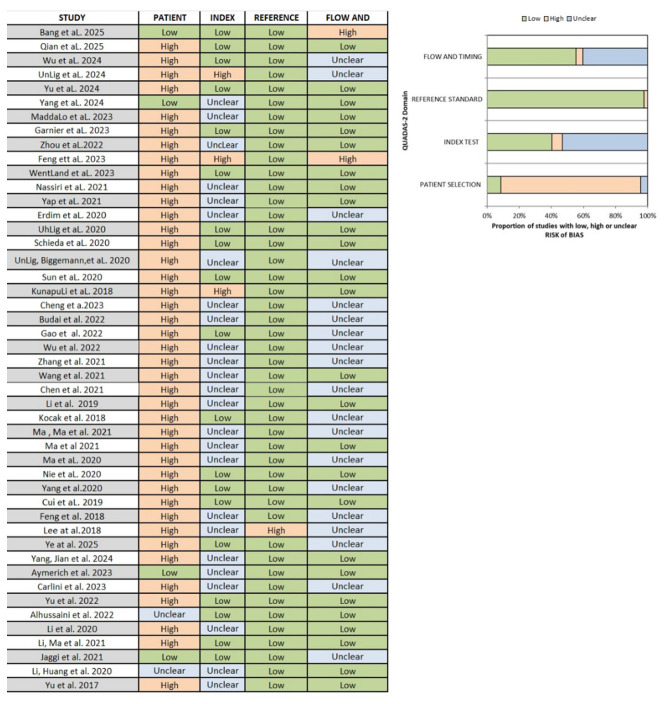
Risk of bias of included studies according to the QUADAS-2 framework [34,35,36,37,38,39,40,41,42,43,44,45,46,47,48,49,50,51,52,53,54,55,56,57,58,59,60,61,62,63,64,65,66,67,68,69,70,71,72,73,74,75,76,77,78,79,80].

**Figure 3:**
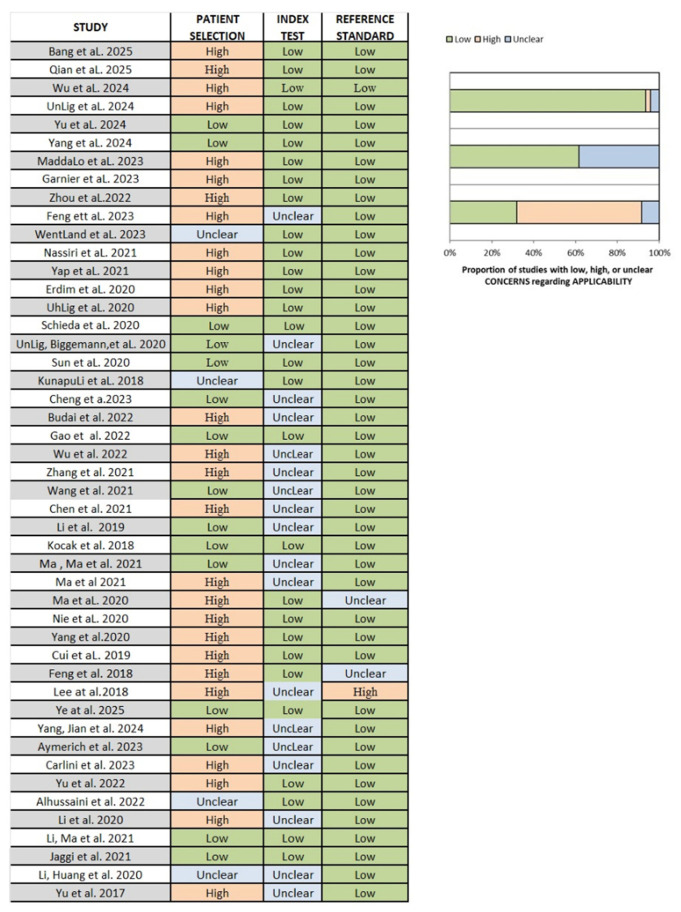
Risk of applicability concerns of included studies according to the QUADAS-2 framework [34,35,36,37,38,39,40,41,42,43,44,45,46,47,48,49,50,51,52,53,54,55,56,57,58,59,60,61,62,63,64,65,66,67,68,69,70,71,72,73,74,75,76,77,78,79,80].

Figure 2 and Figure 3 present the risk of bias and applicability concerns for each individual study, as well as a graphical summary of the QUADAS-2 assessments across all included studies.

### 3.3. Methodological Quality Assessment

**Figure 4:**
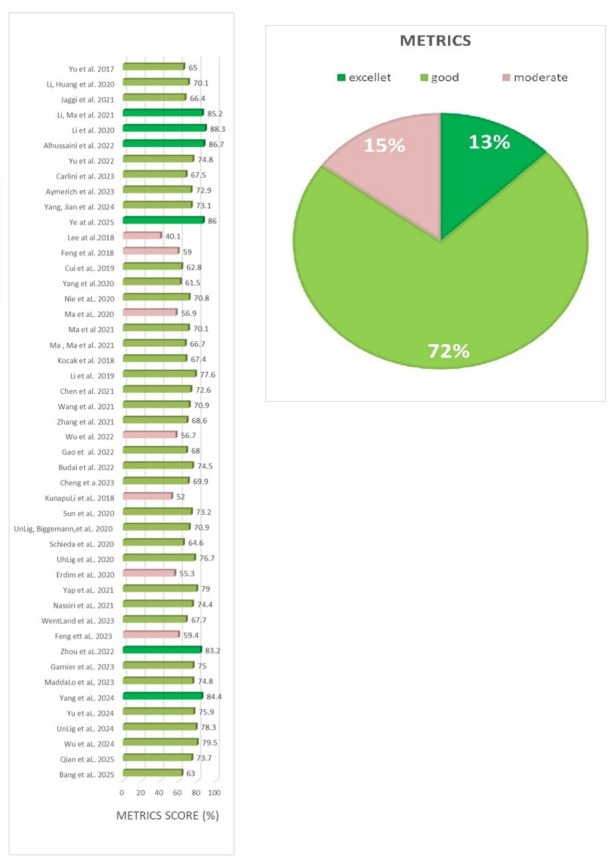
Methodological quality across included studies based on the METRICS [34,35,36,37,38,39,40,41,42,43,44,45,46,47,48,49,50,51,52,53,54,55,56,57,58,59,60,61,62,63,64,65,66,67,68,69,70,71,72,73,74,75,76,77,78,79,80].

Regarding the methodological quality of the radiomics models, overall, it was high, with the majority of studies (n = 40; 85%) categorized as either good (72%) [34,35,36,37,38,39,40,41,44,45,46,48,49,50,51,53,54,55,57,58,59,60,61,62,63,65,67,71,72,73,74,78,79,80] or excellent (13%) [42,66,70,75,76,77]. WEThis distribution indicates a high level of adherence to standardized radiomics reporting and validation protocols. A minority of studies (n = 7; 15%) [43,47,52,56,64,68,69] were classified as having moderate methodological quality, due to limitations in external validation or reporting of image acquisition parameters. Comprehensive scoring details for each individual study, and the methodological quality of the included studies, as evaluated by the METRICS, is shown in Figure 4.

### 3.4. Studies Characteristics

A total of 47 studies were included in our systematic review, encompassing a cumulative cohort of 11,999 patients (mean age: 56.82 ± 13.27 years; male to female [M/F] ratio: 6999/4918). The clinical focus of these studies spanned four primary diagnostic tasks: the differentiation of benign renal tumors from RCC (n = 19, 39.2%) [34,35,36,37,38,39,40,41,42,43,44,45,46,47,48,49,50,51,52]; the discrimination of ccRCC from non-ccRCC subtypes (n = 11, 23.5%) [39,51,53,54,55,56,57,58,59,60,61]; the discrimination of fpAML from RCC (n = 8, 15.7%) [62,63,64,65,66,67,68,69]; and the differentiation of RO from RCC (n = 11, 21.6%) [70,71,72,73,74,75,76,77,78,79,80].

The overall studies characteristics are shown in Table 1, Table 2, Table 3 and Table 4.

### 3.5. CT-Based Radiomics for Differentiating Benign from Malignant Renal Tumors

The studies selected for the current systematic review on the characterization of renal tumors were mainly retrospective (n = 18) [34,35,36,37,38,39,40,41,42,43,44,46,47,48,49,50,51,52], with only one prospective study [45]. Thirteen were conducted in a single institution [34,35,38,40,41,43,44,45,46,47,49,51,52], whereas six were multicenter studies [36,37,39,42,48,50]; one of these also used data from The Cancer Imaging Archive (TCIA) public database [42] (Table 1).

A total of 8007 patients (mean age: 54.4 ± 13.5 years) with solid renal tumors were included. The M/F ratio was reported in 18 studies [34,35,36,37,38,39,40,41,42,43,44,45,46,47,48,49,50,51], comprising 4493 male and 3407 female patients.

Histologic confirmation of renal tumors was reported in all studies. The type of surgery was reported in 15 studies and included nephrectomy, PN, tumorectomy, or biopsy. Malignancy was histologically confirmed in 6281 renal tumors, including 6171 RCCs (ccRCCs: 4658; pRCCs: 845; chRCCs: 602). Benign tumors were histologically confirmed in 1813 cases, including 387 fpAMLs, 651 AMLs, and 599 ROs. The mean tumor size, calculated from seven eligible studies [37,41,43,44,47,48,49], was 4.69 ± 3.15 cm (Table 1).

Overall, all studies reported the intravenous administration of an iodinated contrast medium. The corticomedullary phase (CMP) and nephrographic phase (NP) were predominantly used in CT-based radiomics studies to distinguish between benign and malignant solid renal tumors. Four studies used a four-phase CT protocol, including unenhanced CT (UECT), CMP, NP, and excretory phase (EP) [34,45,46,52]. Eight studies used a three-phase protocol, including UECT, CMP, and NP in four studies [38,42,49,51], and CMP, NP, and EP in four reports [35,36,41,43]. Three studies used two phases, including UECT and CMP in one study [47], and CMP and NP in two studies [37,39]. Finally, three studies used only the NP [40,48,50] (Table S1).

Segmentation strategies are presented in Table 5. Eight studies utilized manual three-dimensional (3D) segmentation of the whole tumor volume [34,35,36,37,40,45,48,50]. To avoid partial volume effects, two reports [43,47] drew boundaries 1 mm inside the tumor margins. Another study also employed manual 3D segmentation, but expanded this to include the intratumoral subregion (5 mm inside the borders) and peritumoral segments (defined as 3 mm and 5 mm beyond the margins, and 6 mm and 8 mm across the tumor boundaries) [38]. One study used manual two-dimensional (2D) segmentation of the tumor’s largest diameter [50], while three studies applied both manual 2D and 3D segmentation [42,46,52]. Semi-automated 3D segmentation of the whole neoplasm was utilized in three reports [41,44,51], and a 3D automated deep learning (DL)-based approach was used in one study [39].

Texture RFs were the most commonly used, often combined with shape and histogram features in nine studies [34,35,37,40,41,42,48,50,51], with shape features in three studies [39,45,46], and with histogram features in three reports [38,44,49]. Transform-based features appeared in four studies, combined with histogram features in one [36], with shape and histogram features in another [43], and with shape-based, first- and second-order statistics in two studies [48,52]. Among texture features, gray-level co-occurrence matrix (GLCM), gray-level run length matrix (GLRLM), and gray-level size zone matrix (GLSZM) were most frequent, followed by neighboring gray-tone difference matrix (NGTDM) and gray-level difference matrix (GLDM) (Table 6).

Statistical screening, reproducibility analysis, and integrated methods, including the Least Absolute Shrinkage and Selection Operator (LASSO), Random Forest (RF), and extreme Gradient Boosting (XGBoost) were the most frequently used approaches for feature selection, with combinations of methods being common to enhance robustness. Specifically, statistical screening was used alone in three studies [34,36,41], combined with reproducibility analysis in two [40,47], and with reproducibility plus performance-based methods (Recursive Feature Elimination-Support Vector Machines, RFE-SVM) in three [37,48,51]. It was also paired with reproducibility and integrated selection methods, including LASSO, in three reports [35,38,43]. Other filter methods included reproducibility analysis alone [45], RFE-SVM [50,52], and integrated approaches such as LASSO [39], RF [44,46], and XGBoost [49] in four studies. Additionally, one study used reproducibility analysis with CatBoost [42] (Table 6).

Six studies used a single ML algorithm, including RF in one study [44], Gradient Boosting in four [37,42,49,52], k-Nearest Neighbors (kNN) in one [40], and Logistic Regression (LR) in one [38] study. Feature selection was applied in two studies: RFE-SVM in one [51] and LASSO combined with SVM, RF, and Decision Tree (DT) in another [41]. The remaining 10 studies employed multiple algorithms, most frequently Gradient Boosting, SVM, and RF [34,35,36,39,43,45,46,47,48,50]. Overall, Gradient Boosting algorithms were the most commonly used (Table 6).

In seven studies, a non-radiomics model, using clinical, and/or conventional CT features, and intratumoral ecological diversity features was also used [35,41,42,43,45,51] (Table S2).

CT-based radiomics signatures demonstrated promising diagnostic accuracy in differentiating benign renal tumors from RCC. Across 19 studies, AUC values ranged from 0.680 to 0.960, yielding a median AUC of 0.830 (IQR: 0.747–0.900). In a subset of four studies [35,38,43,45], the radiomics signature (mean AUC: 0.900 ± 0.054) and combined models (AUC: 0.916 ± 0.058) outperformed clinical models (AUC: 0.741 ± 0.085). The mean AUC improvement was 0.159 for the radiomics signature and 0.174 for the radiomics nomogram compared to the clinical model. The radiomics nomogram demonstrated a small but consistent additional improvement over the radiomics signature (mean difference: 0.0155).

The highest-performing radiomics pipeline was developed to preoperatively differentiate small benign renal masses (≤4 cm)—including typical AMLs, fpAMLs, and rare benign tumors—from RCCs [43]. Utilizing manual 3D segmentation in 146 solid renal tumors, the study extracted 107 original radiomic features and 479 Laplacian of Gaussian (LoG) filtered features. A radiomics nomogram was then constructed by integrating clinical factors (age, sex, and tumor size) with a “Rad-score” derived from the CMP, NP, EP, and combined phase joint. This nomogram achieved an AUC of 0.968 in the internal validation set and effectively distinguished fpAML from RCC with an AUC of 0.946. Comparison of classification techniques revealed that the LR model provided superior discriminative performance over the DT model [43].

### 3.6. CT-Based Radiomics for Differentiating Clear Cell Renal Cell Carcinoma from Non-Clear Cell Renal Cell Carcinoma

Eleven retrospective studies examined the role of CT-based radiomics for differentiating ccRCC from non-ccRCC [39,51,53,54,55,56,57,58,59,60,61]. Eight were single-center studies [51,53,54,55,57,58,59,62], two of which also used public datasets, including the Kidney Tumor Segmentation 2019 Challenge (KiTS19) [54] and The Cancer Genome Atlas (TCGA) [61]; the remaining three were multicenter studies [39,56,60].

A total of 3161 patients (mean age 57.7 ± 12.9 years) were included in this systematic review. The M/F ratio, reported in nine studies, was 1250/662 [53,54,55,56,57,58,59,60,61]. Histology identified 3161 ccRCCs, 348 papillary pRCCs, and 304 chRCCs. Four studies reported the type of surgery performed (nephrectomy, PN, or biopsy). Tumor size was reported in six studies [51,55,57,58,59,61], and ranged from 1.9 cm to 8.8 cm (Table 2).

Considerable heterogeneity exists in CT protocol selection among the included studies (Table S3). Three studies utilized a comprehensive four-phase CT protocol [55,57,59]. Four studies applied three-phase protocols, including UECT, CMP, and NP [51,60], UECT, CMP, and EP [54], and CMP, NP, and EP [58]. Two-phase protocols were reported in three studies (UECT, CMP [61] and CMP, NP [39,56]), while one study used CMP alone [53]. Overall, CMP and NP represent the core phases most commonly utilized across studies.

Manual 3D segmentation was employed in seven studies, including the whole tumor volume [53,60], excluding tumor margins [54], and defined 1–2 mm inside the tumor margins [55,57,58,59]. In one report, manual 2D segmentation of the largest tumor slice was performed, excluding the outer 1–2 mm of the lesion [61]. Finally, a semi-automated 3D segmentation [51] and an automated segmentation of the entire tumor [56] was applied in two studies (Table 7).

Reporting of features used in the radiomics pipeline was included in 10 studies. Texture features were the most frequently used, often combined with shape-based and histogram features in four studies [51,54,56,57], with shape features in one study [39], with histogram features in one report [53], with histogram and transform-based features in one study [61], and with shape, histogram, and transform-based features in another study [51] (Table 8).

Feature selection most commonly combined reproducibility analysis with embedded methods, as seen in five studies using LASSO [57], RF [60], ML algorithms [61], and LASSO with statistical screening [54,57]. Two studies applied only statistical methods and LASSO as filtering approaches [39,52]. Two studies combined statistical screening with reproducibility analysis or LASSO [58,59]. Finally, one study used reproducibility analysis, statistical tests, and RFE-SVM [51] (Table 8).

Five studies employed a single ML algorithm: RFE-SVM [51], LR [55,57], Gradient Boosting [56], LASSO [59], and RF [60]. One study combined SVM with RF [54], while two studies applied SVM in combination with RF and LR [59], and with neural networks (NN) and Gradient Boosting [60]. Two additional studies utilized multiple ML algorithms, including SVM, RF, and Gradient Boosting [39,53] (Table 8).

In six studies, non-radiomics models were also assessed [51,53,55,57,59,60] (Table S2).

Most studies in this systematic review used internal validation, while one utilized an external cohort [54]. The radiomics signature demonstrated excellent diagnostic performance in differentiating ccRCC from non-ccRCC, with AUCs ranging from 0.820 to 0.950 and a median AUC of 0.900 (0.861–0.910), proving highly reliable for clinical decision-making.

In a subgroup of four studies [53,55,59,60], both the radiomics signature (AUC: 0.896 ± 0.057) and the radiomics nomogram (AUC: 0.908 ± 0.056) demonstrated robust diagnostic performance. According to the within-study comparison, the radiomics nomogram consistently outperformed the radiomics signature, with a mean AUC difference of 0.0112.

Across three studies [55,59,60], non-radiomics models utilizing clinical and conventional CT features achieved a mean AUC of 0.777 (± 0.054) for differentiating ccRCC from non-ccRCC. Within the same cohort, radiomics signatures demonstrated superior performance (AUC: 0.866 ± 0.039), with a mean AUC improvement of 0.089.

Among the top-tier radiomics pipelines for differentiating ccRCC from non-ccRCC, one study utilizing only the CMP in a total of 149 RCCs achieved an AUC of 0.929 (0.855–1.000) for the radiomics signature and 0.949 (0.885–1.000) for the combined model [53]. The non-radiomics component integrated clinical data (age, and gender) and morphologic CT findings (size, enhancement, intratumoral vessels, and renal vein invasion). Following manual 3D whole-tumor segmentation, 1168 original and filtered radiomic features were extracted. Among the various ML algorithms tested, LR demonstrated the superior diagnostic performance [53].

### 3.7. CT-Based Radiomics for Differentiating Fat-Poor Angiomyolipoma from Renal Cell Carcinoma

Eight retrospective, single-center studies assessed CT radiomics in distinguishing fpAML from RCC [62,63,64,65,66,67,68,69], three of which were conducted by the same research group [62,63,64]. Six studies focused on differentiating fpAML from ccRCC [62,63,64,65,67,69].

A total of 1021 patients (mean age 55.2 ± 13.0 years; M/F: 386/292) were included, comprising 287 fpAMLs, and 732 RCCs, of which 530 were of the clear cell type. Patients with histologically confirmed fpAMLs showed a female predominance (M/F: 97/166). The mean tumor size, based on data from four eligible studies [63,64,68,69], was 3.1 ± 1.3 cm. The surgical procedures reported in eight studies included nephrectomy and PN (Table 3).

Five studies used a three-phase CT protocol, including UECT, CMP, and NP [62,63,64,66,67], two reported all CT phases [65,66], and one included only the NP (Table S4) [69].

Manual 3D segmentation of renal tumors was performed in five studies: one included the entire lesion [65]; three segmented 2–3 mm within the tumor margins [62,64,67]; and one defined boundaries both 2–3 mm inside and 2 mm outside the lesion [63]. Manual 2D segmentation based on the largest tumor slice was applied in three studies: two included the whole tumor [66,69], and one excluded the outer 2–3 mm (Table 9) [68].

In CT-based radiomics studies distinguishing fpAML from RCC, texture-based features were most commonly used, often combined with histogram and shape features [62,63,64,66,69], histogram features alone [68], or unfiltered and filtered features [65,67]. Feature selection methods included reproducibility analysis with statistical tests and LASSO [62,63,64,65], reproducibility analysis with statistical tests and RFE [68], statistical screening [69], RFE [67], and a 28-feature selection method (Table 10) [66].

Model training approaches included LR [62,63,64], RF [69], RFE-based modeling [67,68], and multiple ML algorithms [66] (Table 10). Four studies also employed non-radiomics models [62,63,65,67] (Table S2).

The radiomics signature across the eight studies included in this review, all using an internal test set, proved highly effective for differentiating fpAML from RCC, yielding a median AUC of 0.912 (0.879–0.933; range: 0.774–0.960). With a median AUC of 0.907 (0.857–0.925) and a performance peak of 0.970, the radiomics signature also demonstrated superior discriminative power between fpAML and ccRCC, based on a subset of six studies [62,63,64,65,67,69]. This high level of accuracy supports the integration of CT radiomics into clinical workflows for improved diagnostic confidence.

High diagnostic performance was observed across three studies differentiating fpAML from ccRCC [62,64,65] for both radiomics models (AUC: 0.898 ± 0.045) and combined models (AUC: 0.968 ± 0.020). In within-study comparisons, the radiomics nomogram consistently outperformed the radiomics signature, with a mean AUC improvement of 0.070.

Across three studies [64,65,67] differentiating fpAML from ccRCC, both clinical and radiomics models achieved high accuracy, with mean AUCs of 0.831 ± 0.134 and 0.914 ± 0.063, respectively. Within-study comparisons varied: the radiomics signature was slightly inferior to clinical models in two reports [64,65] but substantially outperformed the clinical model in one [67], resulting in an overall mean AUC improvement of 0.083.

In a high-performing radiomics framework, researchers utilized triple-phase CT data—including UECT, CMP, and NP—to differentiate fpAML from ccRCC [64]. Following whole-tumor segmentation (defined 2–3 mm within tumor boundaries), logistic classifiers were developed by integrating qualitative conventional CT features, such as angular interface, cyst degeneration, and presence of pseudocapsule, with quantitative radiomics scores. Although conventional analysis alone achieved a robust AUC of 0.935, the inclusion of radiomics significantly improved diagnostic accuracy. Notably, radiomics performance in the UECT group exceeded that of the CMP and NPs, while the “sum group” yielded the highest individual radiomics value. Ultimately, the final combined model achieved a superior AUC of 0.988, demonstrating that the integration of multi-phase radiomics and conventional imaging features optimizes the characterization of solid renal tumors [64].

### 3.8. CT-Based Radiomics for Differentiating Renal Oncocytoma from Renal Cell Carcinoma

Eleven studies evaluated CT-based radiomics for differentiating RO from RCC [70,71,72,73,74,75,76,77,78,79,80], including three from the same authors [76,77,79]. Eight studies focused specifically on distinguishing RO from chRCC [70,71,72,75,76,78,79,80]. Ten studies were retrospective [70,71,72,73,74,76,77,78,79,80], while one included both retrospective and prospective components [75]. Seven were conducted at a single institution [71,72,73,74,78,79,80], and four were multicenter studies [70,75,76,77].

The total population was 1108 patients (mean age 56.45 ± 12.6 years; M/F: 435/389) with 364 histologically confirmed ROs and 679 RCCs, including 468 chRCCs. Nephrectomy, PN, tumor biopsy, and active surveillance were among the surgical reference standards reported in seven studies (Table 4).

The NP was included in all reports. Specifically, one study used a four-phase CT protocol [73], four studies used a three-phase CT protocol (UECT, CMP, and NP [71], CMP, NP, and EP [76,77,79]), one used a two-phase protocol [CMP, and NP [70], and four included only the NP [72,75,78,80] (Table S5).

Manual 3D segmentation was commonly used to delineate renal tumors, including the entire tumor [74,76,77,78,79]; regions within 1 mm of tumor margins [73]; the tumor with its transition zone [73]; and the tumor with peritumoral regions extending 1–3 mm beyond the margins [70]. One study focused on the mid-tumor area across 10 slices [80]. Another combined manual 3D delineation (within 2 mm of lesion boundaries) with semi-automated whole-tumor segmentation [75] (Table 11).

Histogram and texture-based features were most commonly used, often combined with shape and/or transformed features [70,72,73,74,75,76,77,78,79,80]. One study used only high-order features [73] (Table 12).

Various methods were applied for RFs selection. Eight studies used a combination of reproducibility analysis, statistical screening, and LASSO [70,71,72,74,76,77,78,79]. Other methods included LASSO alone [75], SVM [80], and a genetic algorithm [73].

Model training approaches included LR [71,76,77], SVM [70,74,80], DT [73], RF combined with Gradient Boosting [78], and multiple ML algorithms [75,79]. SVM, LR, and RF were the most commonly used ML algorithms applied to differentiate between RO and RCC (Table 12). Four studies also incorporated clinical models [71,74,76,77] (Table S2).

Across the ten studies analyzed [70,71,72,73,76,77,78,79,80], the radiomics signature demonstrated strong diagnostic performance in distinguishing RO from RCC. Utilizing internal test sets in eight studies [70,71,72,73,75,78,79,80], and external validation sets in two reports [76,77], these studies achieved a median AUC of 0.885 (0.841–0.947; range: 0.710–1.000). Furthermore, an eight-study subset [70,71,72,75,76,78,79,80] focusing on the differentiation between RO and chRCC also showed high discriminative power, yielding a median AUC of 0.891 (0.818–0.959) and a peak performance of 1.000.

In a sub-analysis of three studies [74,76,77], radiomics nomograms significantly outperformed clinical models (mean AUC: 0.945 ± 0.045 vs. 0.762 ± 0.133), yielding an average improvement of 0.183. Furthermore, a subset comparison [71,76,77] demonstrated that while radiomics signatures achieved a mean AUC of 0.880 ± 0.066, the integrated nomograms performed better at 0.939 ± 0.045—a consistent intra-study improvement of 0.059.

Employing a robust radiomics pipeline, researchers differentiated ROs (*n* = 47) from chRCCs (*n* = 94) using tri-phasic CECT. A radiomics signature—constructed from 12 optimal features across CMP, NP, and EP—yielded a median AUC of 0.959. By integrating the resulting Rad-score with clinical predictors, specifically segmental enhancement inversion, the team developed a radiomics nomogram. This integrated model achieved a superior AUC of 0.988 in the external validation cohort, outperforming both the standalone clinical model (AUC: 0.895) and the radiomics signature. These findings highlight the nomogram’s potential as a high-fidelity tool for the non-invasive differentiation of histologically similar renal tumors [76].

## 4. Discussion

To the best of our knowledge, this is an up-to-date systematic review evaluating the role of CT-based radiomics in the characterization of solid renal tumors. Specifically, this review focuses on four subcategories: differentiation between benign renal tumors and RCC; RCC histologic characterization (distinguishing ccRCC from non-ccRCC); differentiation between fpAML and RCC; and discrimination between RO and RCC. In total, 47 research studies involving 11,999 patients were evaluated. With a median AUC consistently hovering between 0.830 and 0.912 across all subcategories, CT-based radiomics is proving to be a robust tool for the characterization of solid renal masses. The findings of this review underscore a pivotal shift in renal oncology: the transition from subjective, qualitative image interpretation to objective, data-driven “virtual biopsies.”

Despite the limited number of studies, hybrid models (nomograms)—which integrate the radiomic “Rad-score” with traditional clinical markers and conventional CT findings—outperform standalone radiomic or clinical models. This suggests that radiomics is most effective not as a replacement for clinical judgment, but as a high-fidelity “extension” of it.

By converting standard medical images into high-dimensional, mineable data, CT-based radiomics addresses the critical clinical challenge of differentiating RCC from common benign mimics like fpAML and RO. The radiomics workflow—the transition from raw CT imaging to a predictive clinical model—comprises a sequence of sophisticated computational stages. Initially, image acquisition utilizes standardized CT phases, typically the CMP and NP. This is followed by renal tumor segmentation, where the VOI is delineated; 3D manual or semi-automated segmentation of the entire tumor volume is more often utilized for capturing spatial heterogeneity, offering a “virtual biopsy” advantage.

Traditional renal tumor biopsy is invasive and associated with several limitations, including non-diagnostic sampling, sampling errors related to intratumoral heterogeneity, and procedure-related complications such as bleeding, pain, infection, and, rarely, needle-track tumor seeding. In contrast, radiomics analysis is non-invasive and allows repeated evaluation of the entire tumor volume [1,18,19,20,21,22,23,24,25,26,27,28,29,30,34,35,36,37,38,39,40,41,42,43,44,45,46,47,48,49,50,51,52,53,54,55,56,57,58,59,60,61,62,63,64,65,66,67,68,69,70,71,72,73,74,75,76,77,78,79,80].

During feature extraction, high-dimensional quantitative data are retrieved, including first-order statistics (intensity histograms), shape-based metrics, and advanced texture features. These texture descriptors, such as the GLCM and the GLRLM, quantify spatial distributions and “subtle grains” imperceptible to the human eye. Finally, these features are processed via ML frameworks, employing techniques such as LASSO for dimensionality reduction and algorithms like Gradient Boosting, RF, or SVM for robust classification and predictive modeling [18,19,20,21,22,23,24,25,26,27,28,29,30,34,35,36,37,38,39,40,41,42,43,44,45,46,47,48,49,50,51,52,53,54,55,56,57,58,59,60,61,62,63,64,65,66,67,68,69,70,71,72,73,74,75,76,77,78,79,80].

The differentiation between benign and malignant solid renal tumors remains a cornerstone of personalized urologic oncology, especially given that approximately 20% of surgically resected small renal masses are postoperatively confirmed as benign [2,3,9,10]. Our analysis of 19 studies—encompassing 8007 patients—demonstrates that CT-based radiomics provides a robust, non-invasive “virtual biopsy” to address this clinical challenge. With a median AUC of 0.830, radiomics signatures show high diagnostic performance in identifying RCC.

Our results are in accordance with a previously published systematic review on CT radiomics for differentiating renal tumors, which reported high levels of performance (AUC: 0.82–0.96) [28]. The most striking difference between the two reports is the increase in the evidence base (from eight to 47 studies in our review), indicating that radiomics has evolved from a niche experimental interest into a robust field of oncological research.

Both reviews noted that multi-phase CT is superior to single-phase analysis for the characterization of solid renal tumors. In our review, the studies exhibited a preference for the CMP and NPs, which are optimal for capturing the differential enhancement patterns between RCC and benign mimics, such as fpAML and RO. The previous review noted that all 13 studies relied on manual tumor segmentation, characterizing it as a major limitation due to a lack of reproducibility and extreme time cost. While manual 3D segmentation is still considered the “gold standard”, the current review includes studies utilizing automated DL-based and semi-automated approaches. Automated and semi-automated segmentation techniques overcome the limitations of manual methods by providing standardized reproducibility, high-throughput efficiency, and voxel-level precision, effectively transforming 3D radiomics from a labor-intensive task into a scalable, mathematically rigorous clinical tool [81,82].

Both reviews underline that texture analysis remains the most powerful radiomic feature for renal applications. Texture analysis provides a quantitative bridge between visual imaging and underlying renal tumor biology by mathematically capturing the spatial distribution of pixel intensities (heterogeneity) to differentiate between benign and malignant lesions, with a level of objectivity unattainable by human visual inspection alone. In addition, the current review shows a richer feature landscape, with more frequent use of LoG filters, and transform-based features. Filtered radiomics features enhance the assessment of renal tumors by mathematically deconstructing images to isolate multiscale heterogeneity and suppress imaging noise, thereby uncovering hidden spatial patterns that improve the diagnostic accuracy and reproducibility of tumor subtype differentiation [81,82].

Regarding the ML algorithms, our review addresses a shift from SVMs—reported as the most common method in the previous study—toward Gradient Boosting algorithms and RF. This suggests a move toward more complex ensemble models that handle high-dimensional radiomic data and non-linear relationships, offering superior predictive stability and built-in feature importance rankings compared to simpler linear models [81,82].

Differentiating fpAML from RCC—particularly the clear cell subtype—is a notorious CT challenge, which often leads to “overtreatment” [3,9,10,83,84,85,86]. In our analysis of eight studies (1021 patients), CT-based radiomics demonstrated high diagnostic efficacy, with an AUC of 0.912 in differentiating fpAML from RCC, and an AUC of 0.907 in distinguishing between fpAML and ccRCC.

Our results are highly congruent with a previous meta-analysis, including 10 studies (1456 lesions), which reported pooled specificities exceeding 92% across both subgroups [29]. Both studies suggest that radiomics is exceptionally reliable at “ruling in” fpAML—meaning that if the model predicts fpAML, there is a very high probability the lesion is indeed benign.

A significant finding across both our review and the existing literature is the critical role of the UECT phase, which appears to capture the internal texture and “gray-level” distribution of fpAML [29,64]. In addition, our analysis suggests that the current State-of-the-Art involves multi-phase integration, with combined models achieving AUCs as high as 0.988, potentially offering a definitive non-invasive solution to the clinical dilemma of the “fat-poor” renal mass. This suggests that while UECT is the strongest individual phase, modern ML algorithms are now better at “stacking” data from multiple phases to improve accuracy [64].

The “gray zone” between RO and RCC represents one of the most difficult distinctions in renal pathology. Renal oncocytoma has variable and nonspecific CT findings that overlap with the characteristics of chromophobe RCC. Both tumors originate from the intercalated cells of the collecting ducts and share nearly identical morphological and imaging features [3,9,10,87,88,89,90].

Based on our review, across a cohort of 1108 patients from 11 different studies, CT-based radiomics exhibited robust diagnostic performance in distinguishing between these two entities. The median AUC reached 0.885 in the discrimination of RO from RCC, while the differentiation between RO and chRCC yielded an AUC of 0.891, confirming that radiomics is uniquely suited for the RO vs. chRCC differential, a task that remains a significant diagnostic pitfall for conventional qualitative CT interpretation. The NP was identified as the core phase for this differentiation, often supplemented by manual delineation of the renal tumor, including the whole tumor and/or the peritumoral transition zone analysis. Texture and histogram features were commonly used, and SVM, LR, and RF algorithms had the best diagnostic performance. By employing SVM and LASSO for feature selection, several studies reached peak performances (AUC: 1.00, in small cohorts), highlighting that radiomics can detect micro-architectural differences that are often indistinguishable even on high-quality conventional CECT [75].

Our findings align with the pooled results of a previous meta-analysis, including six studies with 1064 lesions, which reported a pooled sensitivity and specificity of 0.83 and 0.92, respectively, for differentiating RO from chRCC [30]. Our expanded cohort of 11 studies suggests a trajectory of increasing diagnostic accuracy, likely driven by the adoption of multi-phase analysis, more sophisticated feature selection techniques, and nomograms. This provides a compelling argument for the use of CT-based radiomics as a supportive tool to avoid biopsy or surgery for suspected renal oncocytomas.

Histologic characterization of RCC is essential for precision management, as main subtypes possess distinct molecular drivers and clinical trajectories. Accurate identification informs prognosis and dictates the choice of appropriate systemic therapy. Furthermore, subtype differentiation guides surgical planning—favoring nephron-sparing approaches for indolent lesions—and identifies patients at risk for hereditary syndromes [91]. The current review of 11 studies (3161 patients) found that CT radiomics achieves excellent discriminative performance (AUC: 0.900). The most effective pipelines utilized multi-phase CT data, particularly the CMP, to capture the characteristic hyper-enhancement of ccRCC. While manual 3D segmentation remained the standard, the integration of shape-based and texture features into LR and Gradient Boosting models provided a superior diagnostic yield over conventional CT characteristics. These findings suggest that CT radiomics can reliably predict the high-risk ccRCC phenotype non-invasively.

Despite the limited number of studies, the current review shows that across all primary diagnostic tasks radiomics signatures consistently outperformed traditional clinical models. While clinical models typically achieved moderate accuracy (AUCs ranging from 0.741 to 0.831), radiomics signatures provided a substantial diagnostic leap. In addition, the radiomics nomogram emerged as the most robust tool. While the radiomics signature itself is highly effective, the nomogram provided a small but consistent additional improvement in predictive performance. The data suggests that the highest diagnostic performance is achieved when AI-driven data is used as a high-fidelity extension of clinical judgment rather than a replacement. By “stacking” multi-phase CT radiomics with clinical factors and/or conventional CT features, these combined models reach peak performances—such as the AUC of 0.968 observed in differentiating fpAML from ccRCC—effectively bridging the gap between qualitative radiology and invasive pathology.

### 4.1. Quality Assessment

Methodological evaluation of the 47 studies included in the current review using the QUADAS-2 tool revealed a significant disparity between technical diagnostic accuracy and overall study design. While internal validity was bolstered by a robust reference standard—with 98% of studies demonstrating low risk of bias in “gold standard” application—systemic vulnerabilities were identified in patient selection, where 87% of studies exhibited a high risk of bias due to non-consecutive sampling or inappropriate exclusions. Furthermore, external validity remains a concern, as 60% of studies raised significant applicability issues regarding their cohorts. Consequently, while the reported diagnostic performance is technically well-grounded in its index and reference standards, the pervasive selection bias and limited representativeness necessitate caution when generalizing these findings to broader clinical populations.

Evaluation of the included studies using the METRICS framework underscores a high degree of scientific reliability, with 85% of the literature categorized as “good” to “excellent.” This suggests that the majority of current radiomics research in this domain utilizes robust data partitioning and maintains high methodological transparency. Notably, the 13% of studies achieving an “excellent” rating represent the highest tier of evidence, often incorporating external validation or open-source practices to mitigate “black box” risks. However, 15% of the studies exhibited “moderate” quality, typically due to limitations such as potential overfitting from small sample sizes, a lack of independent validation cohorts, or insufficient detail regarding image preprocessing and normalization. Overall, while minor methodological gaps persist, the collective evidence demonstrates a strong foundational rigor in radiomics-based renal mass characterization.

### 4.2. Clinical Implications and Future Directions

This systematic review underscores the role of CT-based radiomics as a robust “virtual biopsy” capable of addressing the historical limitations of qualitative image interpretation. Clinically, this diagnostic precision offers a robust pathway to reduce the overtreatment of benign renal tumors, which are currently surgically resected at a rate of approximately 20%. Ultimately, the integration of multi-phase CT radiomics into the urological workflow might provide a scalable, non-invasive tool for precise histologic characterization, ensuring that surgical and systemic interventions are tailored to the specific biological profile of each tumor.

However, despite promising results, the “clinical readiness” of radiomics is currently limited by a need for prospective, multicenter trials and standardized image acquisition protocols. One of the most promising solutions to CT scanners variability is image harmonization via algorithms such as ComBat, which effectively eliminates scanner-specific statistical signatures to enable the pooling of multicenter data [92,93]. Beyond static snapshots, delta-radiomics offers a longitudinal approach by tracking feature evolution over time, providing a superior method for distinguishing pseudo-progression from true tumor growth during immunotherapy [94,95,96]. Furthermore, the emergence of radiogenomics seeks to achieve a “virtual biopsy” by correlating imaging phenotypes with underlying genetic mutations [97,98]. To ensure seamless clinical integration, these advancements are being paired with automated “zero-click” workflows powered by convolutional neural networks that handle everything from automated segmentation to the generation of standardized “Rad-scores” in radiology reports [99,100].

### 4.3. Limitations

This systematic review has some inherent limitations. A major shortcoming is the lack of standardization across the radiomics pipeline; variations in CT acquisition parameters, phase variability, along with divergent segmentation strategies, introduce substantial technical noise that undermines the reproducibility of radiomic features. Methodologically, the QUADAS-2 assessment revealed a high risk of selection bias in 87% of the literature, largely due to retrospective designs and non-consecutive patient sampling, which may lead to an overestimation of model accuracy. Furthermore, while the cumulative cohort is large, many within-study comparisons between radiomics and clinical models rely on small subsets, and there remains a critical shortage of external validation in independent, multicenter populations. The large number of studies lacking a reported definitive reference standard represents another key limitation and may reduce the overall strength of the evidence. Finally, the “black box” nature of complex ML algorithms like Gradient Boosting and SVM often lacks the biological interpretability required for urologists to confidently base operative decisions on a numerical “Rad-score” rather than traditional pathology. The future of renal radiomics should focus on achieving higher reproducibility. By adopting standardized acquisition protocols, automated segmentation, and multicenter external validation, CT-based radiomics might prove a rigorous tool for personalized urologic care.

## 5. Conclusions

CT-based radiomics represents a transformative advancement in the non-invasive characterization of solid renal tumors. By extracting high-dimensional features imperceptible to the human eye, these pipelines provide a repeatable “virtual biopsy” that captures the full spectrum of tumor heterogeneity. This approach offers high diagnostic accuracy in distinguishing malignant from benign renal tumors, thereby reducing overtreatment. Furthermore, the identification of RCC histologic subtypes (ccRCC vs. non-ccRCC) is achievable, which is critical for personalized therapeutic planning. While results are promising, the lack of standardization in CT protocols and the predominance of retrospective data remain significant hurdles. Future research must prioritize prospective, multicenter trials and the development of “scanner-agnostic” algorithms to ensure seamless integration into clinical workflows.

## Figures and Tables

**Table 1:** Baseline characteristics of the included studies for the differential diagnosis of benign renal tumors and renal cell carcinoma.

Study	Year	Type of Study	Outcome	Number of Patients with Renal Tumors	Age (Years)	Sex (Male vs. Female)	Tumor Size (cm)	Type of Surgery
Bang et al. [34]	2025	retrospective, single-center	Dd RCC vs. benign renal tumors (SRMs)	499 (373 RCCs: 200 ccRCCs, 89 pRCCs, 84 chRCCs; 126 benign: 74 fpAMLs, 48 ROs)	56.02 ± 12.18	285/214	3.515 ± 2.42	nephrectomy, PN
Qian et al. [35]	2025	retrospective, single-center	Dd RCC vs. benign malignant tumors	122 (75 malignant: 58 RCCs, 17 various; 47 benign: 40 AMLs, 7 ROs)	54.2 (malignant) 46.5 (benign)	82/40	n/a	n/a
Wu et al. [36]	2024	retrospective, multicenter	Dd RCC vs. benign renal tumors	427 (279 RCCs: 237 ccRCCs, 16 pRCCs, 26 chRCCs; 148 benign: 144 AMLs, 4 ROs)	51 (42–61)	213/214	4.0 (2.8–5.5)	n/a
Uhlig et al. [37]	2024	retrospective, multicenter	renal tumor subtypes	418 (259 ccRCCs, 100 pRCCs, 26 chRCCs, 19 AMLs, 54 ROs)	64 ± 13	268/159	4.2 ± 1.6	nephrectomy, PN
Yu et al. [38]	2024	retrospective, single-center	Dd malignant vs. benign renal tumors	1795 (1396 malignant: 1191 ccRCCs, 51 pRCCs, 78 chRCCs, others; 399 benign (307 AMLs, 18 ROs, others)	56 (18–86, malignant) 50 (18–84, benign)	978/418	3.5 (0.9–13.9, malignant) 3.9 (0.9–19.3, benign)	PN
Yang et al. [39]	2024	retrospective, multicenter	Dd RCC vs. benign renal tumors	1051 (901 RCCs: 678 ccRCCs, 149 pRCCs, 74 chRCCs; 150 benign: 67 AMLs, 23 ROs, 30 others)	52.89 ± 13.94	624/427	n/a	nephrectomy, PN
Maddalo et al. [40]	2023	retrospective, single-center	Dd RCC vs. benign renal tumors (SRMs)	85 (51 RCCs: 37 ccRCCs, 7 pRCCs, 7 chRCCs; 34 benign: 7 fpAMLs, 25 ROs, 2 leiomyomas)	67 (RCC), 64 (benign)	35/16 (RCC), 16/18 (benign)	28.5 (RCC), 22.6 (benign)	nephrectomy, PN, tumorectomy
Garnier et al. [41]	2023	retrospective, single-center	Dd RCC vs. benign renal tumors	122 (111 RCCs: 79 ccRCCs, 13 pRCCs, 16 chRCCs, 3 rare; 21 benign: 2 fpAMLs, 18 ROs, 1 rare)	58 ± 14	87/45	4.3 (1–12.3)	PN
Zhou et al. [42]	2023	retrospective, multicenter + TCIA	Dd RCC vs. benign renal tumors	798 (680 RCCs: 533 ccRCCS, 78 pRCCs, 69 chRCCs; 125 benign: 83 fpAMLs, 42 ROs)	53.88 ± 12.24 (benign) 56.35 ± 12.66 (RCC)	160/638	n/a	surgery, biopsy
Feng et al. [43]	2023	retrospective, single-center	Dd RCC vs. benign renal tumors (SRMs)	156 (92 RCCs: 79 ccRCCs, 7 pRCCs, 6 chRCCs; 64 benign: 37 AMLs, 25 fpAMLs, 1 RO, 1 rare)	54.65 ± 12.12 (RCC) 44.36 ± 11.66 (benign)	70/86	31.19 ± 8.04 (RCC) 25.85 ± 8.91 (benign)	n/a
Wentland et al. [44]	2023	retrospective, single-center	Dd RCC vs. benign renal tumors	148 (98 RCCs: 23 ccRCCs, 44 pRCCs, 31 chRCCs; 50 benign: 23 AMLs, 27 ROs)	57.5 ± 12.1 (25–87)	87/61	3.3 ± 1.6 (1.2–11.6, RCC) 2.7 ± 1.1 (1.2–5.6, benign)	PN
Nassiri et al. [45]	2022	prospective, single-center	Dd RCC vs. benign renal tumors	684 (521 RCCs: 401 ccRCCs, 73 pRCCs, 42 chRCCs, 5 others; 163 benign: 59 fpAMLs, 104 ROs)	62 (23–94, RCC) 63 (17–92, benign)	468/215	<4: 284/106; 4–7: 149/32; 7–10: 48/12; >10: 40/13	nephrectomy, PN
Yap et al. [46]	2021	retrospective, single-center	Dd RCC vs. benign renal tumors	735 (539 malignant: 407 ccRCCs, 73 pRCCs, 42 chRCCs, 17 others; 196 benign (59 fpAMLs, 104 ROs, 33 others)	60.8 (17–93)	495/240	n/a	nephrectomy, PN
Erdim et al. [47]	2020	retrospective, single-center	Dd RCC vs. benign renal tumors	79 (63 RCCs: 25 ccRCCs, 23 pRCCs, 15 chRCCs; 22 benign: 11 fpAMLs, 10 ROs)	57.2 ± 12.6 (RCC) 54.9 ± 15.5 (benign)	55/24	58.41 ± 33.01 (RCC) 36.04 ± 13.96 (benign)	surgery, biopsy
Uhlig et al. [48]	2020	retrospective, multicenter	Dd RCC vs. benign renal tumors	201 (171 RCCs: 131 ccRCCs, 29 pRCCs, 11 chRCCs; 30 benign: 14 AMLs, 16 ROs)	66 (56–74)	121/73	5.16 (3.84–6.84)	nephrectomy, biopsy
Schieda et al. [49]	2020	retrospective, single-center	Dd RCC vs. benign renal tumors	165 (116 RCCs: 51 ccRCCs, 40 pRCCs, 25 chRCCs; 61 benign: 12 fpAMLs, 49 ROs)	52 ± 16 62 ± 10 (benign) 63 ± 12 59 ± 12 (RCC)	36/25 (benign) 36/25 (RCC)	1.9 ± 1.3 (8–44) 3.5 ± 2.4 (6–10.8, benign) 5.2 ± 2.7 (1.7–14.8) 3.6 ± 2.2 (1.2–9.8) 4.5 ± 2.0 (1.7–8.8, RCC)	nephrectomy, biopsy
Uhlig et al. [50]	2020	retrospective, multicenter	Dd RCC vs. benign renal tumors (clinical T1)	94 (76 RCCs: 67 ccRCCs, 7 pRCCs, 2 chRCCs; 18 benign: 9 AMLs, 9 ROs)	64.4	66/28	4,65	nephrectomy, PN
Sun et al. [51]	2020	retrospective, single-center	Dd RCC vs. benign renal tumors	288 (254 RCCs: 190 ccRCCs, 26 pRCCs, 38 chRCCs; 36 benign: 26 fpAMLs, 10 ROs)	55 (19–85)	178/110	3.94	nephrectomy, PN, biopsy
Kunapuli et al. [52]	2018	retrospective, single-center	Dd RCC vs. benign renal tumors	150 (100 RCCs: 70 ccRCCs, 20 pRCCs, 10 chRCCs; 50 benign: 20 fpAMLs, 30 ROs)	n/a	n/a	n/a	n/a

TCIA: The Cancer Imaging Archive; Dd: differential diagnosis; RCC: renal cell carcinoma; SRMs: small renal masses; ccRCC: clear cell renal cell carcinoma; pRCC: papillary renal cell carcinoma; chRCC: chromophobe renal cell carcinoma; fpAML: fat-poor angiomyolipoma; RO: renal oncocytoma; n/a: non-applicable; PN: partial nephrectomy.

**Table 2:** Baseline characteristics of the included studies for the differential diagnosis of clear cell renal cell carcinoma and non-clear cell renal cell carcinoma.

Study	Year	Type of Study	Outcome	Number of Patients with Renal Tumors	Age (Years)	Sex (Male vs. Female)	Tumor Size (cm)	Type of Surgery
Yang et al. [39]	2024	retrospective, multicenter	Dd ccRCC vs. non-ccRCC	901 RCCs (678 ccRCCs, 149 pRCCs, 74 chRCCs)	52.89 ± 13.94	n/a	n/a	nephrectomy, PN
Sun et al. [51]	2020	retrospective, single-center	Dd ccRCC vs. non-ccRCC	254 (190 ccRCCs, 26 pRCCs, 36 chRCCs)	59 (23–85, ccRCC) 54 (19–76, pRCC) 51 (24–83, chRCC)	n/a	4.00 (ccRCC) 4.16 (pRCC) 4.61 (chRCC)	nephrectomy, PN, biopsy
Cheng et al. [53]	2023	retrospective, single-center	Dd ccRCC vs. non-ccRCC	147 (100 ccRCCs, 25 pRCCs, 22 chRCCs)	58.56 ± 11.42 (29–80, ccRCC) 53.06 ± 13.37 (31–82 non-ccRCC)	67/33 (ccRCC) 31/16 (non-ccRCC)	n/a	n/a
Budai et al. [54]	2022	retrospective, single-center + KiTS19	Dd ccRCC vs. non-ccRCC	278 (211 ccRCCs, 47 pRCCs, 27 chRCCs)	60.5 (ccRCC) 53 (non-ccRCC)	140/44	n/a	nephrectomy, PN
Gao et al. [55]	2022	retrospective, single-center	Dd RCC subtypes: high-grade ccRCC vs. pRCC 2	142 (71 ccRCCs, 71 pRCCs)	54 (50–66, ccRCC) 61 (55–71, pRCC)	109/33	6.4 (4.1–8.3, ccRCC) 5.5 (3.4–7.5, pRCC)	n/a
Wu et al. [56]	2022	retrospective, multicenter	Dd ccRCC vs. non-ccRCC	443 (350 ccRCCs, 39 pRCCs, 30 chRCCs, 24 others)	59.4 ± 13.5	278/165	n/a	n/a
Zhang et al. [57]	2021	retrospective, single-center	Dd RCC subtypes + ccRCC vs. non-ccRCC	261 (209 ccRCCs, 25 pRCCs, 29 chRCCs)	52 (ccRCC) 52 (pRCC) 54 (chRCC)	166/95	4.507 (ccRCC) 4.523 (pRCC) 4.737 (chRCC)	n/a
Wang et al. [58]	2021	retrospective, single-center	Dd ccRCC vs. non-ccRCC	190 (147 ccRCCs, 24 pRCCs, 13 chRCCs, 6 collecting duct carcinomas)	59.32 ± 10.68 (27–88)	100/90	2–11 (6.5 ± 3.5)	n/a
Chen et al. [59]	2021	retrospective, single-center	Dd ccRCC vs. non-ccRCC	197 (143 ccRCCs, 25 pRCCs, 29 chRCCs)	53.202 ± 13.086 (ccRCC), 52.804 ± 12.857 (non-ccRCC)	123/74	5.283 ± 2.602 cm (ccRCC), 4.929 ± 2.615 (non-RCC)	nephrectomy, PN, biopsy
Li et al. [60]	2019	retrospective, multicenter	Dd ccRCC vs. non-ccRCC	255 (188 ccRCCs, 36 pRCCs, 31 chRCCs)	58.89 (33–81)	169/86	n/a	n/a
Kocak et al. [61]	2018	retrospective, single-center + TCIA	Dd RCC subtypes + ccRCC vs. non-ccRCC	93 (61 ccRCCs, 20 pRCCs, 13 chRCCs)	55.1 (38–88)	67/26	6.85 (2.1–22.6)	n/a

KiTS19: Kidney Tumor Segmentation Challenge 2019; non-ccRCC: non-clear cell renal cell carcinoma.

**Table 3:** Baseline characteristics of the included studies for the differential diagnosis of fat-poor angiomyolipoma and renal cell carcinoma.

Study	Year	Type of study	Outcome	Number of Patients with Renal Tumors	Age (Years)	Sex (Male vs. Female)	Tumor Size (cm)	Type of Surgery
Ma et al. [62]	2021	retrospective, single-center	Dd fpAML vs. ccRCC	139 (29 fpAMLs, 110 ccRCCs)	47.3 ± 10.9 (fpAML) 59.5 ± 12 (ccRCC)	12/17 (fpAML) 77/33 (ccRCC)	n/a	nephrectomy, PN
Ma et al. [63]	2021	retrospective, single-center	Dd fpAML vs. ccRCC	230 (58 fpAMLs, 172 ccRCCs)	48.4 ± 11.9 (fpAML) 61.3 ± 13.2 (ccRCC)	38/20 (fpAML) 59/113 (ccRCC)	2.71 ± 1.55 (fpAML) 3.49 ± 1.29 (ccRCC)	nephrectomy, PN
Ma et al. [64]	2020	retrospective, single-center	Dd fpAML vs. ccRCC	84 (22 fpAMLs, 62 ccRCCs)	50.5 ± 12.8 (fpAML) 57.9 ± 10.8 (ccRCC)	6/16 (fpAML) 38/24 (ccRCC)	3.20 ± 0.98 (fpAML) 3.74 ± 1.56 (ccRCC)	nephrectomy, PN
Nie et al. [65]	2020	retrospective, single-center	Dd fpAML vs. ccRCC	99 (36 fpAMLs, 63 ccRCCs)	50.08 ± 8.3 (fpAMLs) 58.57 ± 11.45 (ccRCC)	10/42 (fpAML) 26/21 (ccRCC)	2.19 (0.78–8.83, fpAMLs) 2.72 (1.30–6.24, ccRCC)	n/a
Yang et al. [66]	2020	retrospective, single-center	Dd fpAML vs. RCC	163 (45 fpAMLs, 113 RCCs: 95 ccRCCs, 10 pRCCs, 13 chRCCs)	48.6 ± 13.7 (fpAML) 52.9 ± 13.1 (RCC)	13/32 (fpAML) 87/31 (RCC)	2.5 (2.1–3.3 fpAML) 2.9 (2.4–3.3, RCC)	nephrectomy, PN
Cui et al. [67]	2019	retrospective, single-center	Dd fpAML vs. RCC	168 (41 fpAMLs, 130 RCCs: 82 ccRCCs, 22 pRCCs, 26 chRCCs)	48.56 ± 12.9 (fpAML) 55.27 ± 11.56 (ccRCC) 49.27 ± 12.99 (pRCC) 55.00 ± 11.8 (chRCC)	11/29 (fpAMLs) 72/56 (RCCs)	<4	nephrectomy, PN
Feng et al. [68]	2018	retrospective, single-center	Dd fpAML vs. RCC (SRMs)	58 (17 fpAMLs, 41 RCCs)	48.7 ± 10.8 (fpAML) 56.2 ± 12.3 (RCC)	7/10 fpAML 27/14 RCC	≤4	nephrectomy, PN
Lee et al. [69]	2017	retrospective, single-center	Dd fpAML vs. ccRCC (SRMs)	80 (39 fpAMLs, 41 ccRCCs)	n/a	n/a	1.62 ± 0.53 (fpAML) 2.36 ± 0.72 (ccRCC)	n/a

**Table 4:** Baseline characteristics of the included studies for the differential diagnosis of renal oncocytoma and renal cell carcinoma.

Study	Year	Type of Study	Outcome	Number of Patients with Renal Tumors	Age (Years)	Sex (Male vs. Female)	Tumor Size (cm)	Type of Surgery
Ye et al. [70]	2025	retrospective, multicenter	Dd RO vs. chRCC	92 (41 ROs, 51 chRCCs)	54.5 ± 8.96 (RO) 51.4 ± 13.63 (chRCC)	38/54	n/a	nephrectomy, PN
Yang et al. [71]	2024	retrospective, single-center	Dd RO vs. chRCC	96 (30 ROs, 66 chRCCs)	53.23 ± 9.71 (RO) 50.82 ± 12.10 (chRCC)	45/51	3.6 (2.35–4.45, RO) 4.3 (3.27–6.00 chRCC)	n/a
Aymerich et al. [72]	2023	retrospective, single-center	Dd RO vs. chRCC	38 (19 ROs, 19 chRCCs)	69 (57–86, RO) 70 (38–85, chRCC)	19/19	n/a	n/a
Carlini et al. [73]	2023	retrospective, single-center	Dd RO vs. ccRCC (SRMs)	77 (30 ROs, 47 ccRCC)	n/a	n/a	n/a	PN
Yu et al. [74]	2022	retrospective, single-center	Dd RO vs. RCC (chRCC, ccRCC)	180 (41 ROs, 139 RCCs: 75 chRCCs, 64 ccRCCs)	59.4 ± 7.8 (RO) 57.3 ± 9.7 (chRCC) 54.0 ± 10.9 (ccRCC)	86/94	n/a	nephrectomy, PN, active surveillance
Alhussaini et al. [75]	2022	prospective + retrospective, multicenter	Dd RO vs. chRCC	78 (41 ROs, 37 chRCCs)	68.17 ± 8.74 (RO) 56.83 ± 14.30 (chRCC)	38/40	3.54 ± 1.47 (RO) 5.4 ± 3.32 (chRCC)	nephrectomy, biopsy
Li et al. [76]	2022	retrospective, multicenter	Dd RO vs. chRCC	141 (47 ROs, 94 chRCCs)	57.16 ± 11.37 (RO) 54.53 ± 11.07 (chRCC)	61/80	n/a	n/a
Li et al. [77]	2021	retrospective, multicenter	Dd RO vs. ccRCC (SRMs)	122 (46 ROs, 76 ccRCCs)	54.43 ± 16.19 (RO) 54.0 ± 10.9 (ccRCC)	21/25 (RO) 64/22 (ccRCC)	≤4	nephrectomy, PN
Jaggi et al. [78]	2021	retrospective, single-center	Dd RO vs. chRCC	102 (42 ROs, 60 chRCCs)	63 ± 12	68/34	n/a	nephrectomy, PN
Li et al. [79]	2020	retrospective, single-center	Dd RO vs. chRCC	61 (17 ROs, 44 chRCCs)	54.9 (35–79, RO) 50.8 (22–79, chRCC)	40/21	n/a	nephrectomy, PN
Yu et al. [80]	2017	retrospective, single-center	Dd RO vs. RCC	119 (10 ROs, 46 ccRCCs, 41 pRCCs, 22 chRCCs)	n/a	n/a	n/a	n/a

**Table 5:** Segmentation strategies used to differentiate benign renal tumors from renal cell carcinoma.

Study	Segmentation Method
Bang et al. [34]	whole tumor, manual 3D segmentation, 2 radiologists
Qian et al. [35]	whole tumor, manual 3D segmentation, 2 radiologists, 3D Slicer (version 5.3.0, https://www.slicer.org/, accessed on 23 May 2026)
Wu et al. [36]	whole tumor, manual 3D segmentation, 4 observers, ITK-SNAP, 3.8.0
Uhlig et al. [37]	3D Slicer
Yu et al. [38]	manual 3D segmentation: whole tumor + 3 mm inside + 3 mm + 5 mm expanding tumor margins + 3 mm + 5 mm peritumoral + 6 mm + 8 mm crossing tumor borders (CMP), whole tumor (UECT, NP), ITK-SNAP
Yang et al. [39]	manual delineation, 3 radiologists + automated kidney and kidney tumor segmentation, 3D-Unet
Maddalo et al. [40]	whole tumor, manual 3D segmentation, 3 radiologists, 3D Slicer, version 4.10.2
Garnier et al. [41]	whole tumor, 3D semi-automated segmentation, NP, SOPHiA DDM, Radiomics v2.1.21 (SOPHiA GENETICS, Saint-Sulpice, Switzerland)
Zhou et al. [42]	manual 2D (largest tumor slice) + 3D (whole tumor) segmentation, 5 radiologists, Python (version 3.6.5), PyRadiomics
Feng et al. [43]	whole tumor, 1 mm from tumor margins, manual 3D segmentation, 3D Slicer, version: 4.10.2.
Wentland et al. [44]	whole tumor, 3D semi-automated segmentation, 2 radiologists, syngoVia Frontier (Siemens Healthineers, Forchheim, Germany)
Nassiri et al. [45]	whole tumor, manual 3D segmentation, 4 radiologists, Synapse 3D (FujiFilm, Stamford, CT, USA)
Yap et al. [46]	whole tumor, manual 2D + 3D segmentation, 4 radiologists, Synapse 3D (Fujifilm)
Erdim et al. [47]	whole tumor, 1mm from tumor margins, manual 3D segmentation, 2 radiologists, MaZda (version 4.6, P. M. Szczypinski, Institute of Electronics, Technical University of Lodz)
Uhlig et al. [48]	whole tumor, manual 3D segmentation, 3D Slicer, PyRadiomics
Schieda et al. [49]	largest tumor slice, manual 2D segmentation, 2 radiologists, ImageJ ^®^, version 1.52r (National Institutes of Health, USA http://rsbweb.nih.gov/, accessed on 23 May 2026)
Uhlig et al. [50]	whole tumor, manual 3D segmentation, 2 radiologists, 3D Slicer
Sun et al. [51]	whole tumor, 3D semi-automated segmentation, 2 radiologists, Python (version 3.6.1, Python Software Foundation)
Kunapuli et al. [52]	manual 2D (largest tumor slice) + 3D (whole tumor) segmentation, 3D Synapse (Fujifilm, Stamford CT)

3D: three-dimensional; 2D: two-dimensional; NP: nephrographic phase; CMP: corticomedullary phase; UECT: unenhanced CT.

**Table 6:** Radiomics pipeline for the differentiation of benign renal tumors from renal cell carcinoma, including high-throughput radiomics features, optimal feature selection methods, machine learning algorithms, and final classification results.

Study	Extracted Features	Feature Selection	Model Training	Classification Results	AUC (±SD OR 95% CI)
Bang et al. [34]	1288 RFs: first order, 3D shape, GLCM, GLRM, GLSZM, NGTDM, GLDM, PyRadiomics + Python (version 3.10.8)	statistical tests + dimensionality reduction	LinearSVM, RadialbasisfunctionSVM, RF, XGBoost	radiomics: XGBoost trained with 20% of principal components + all CT phases	0.744 ± 0.004
Qian et al. [35]	322 RFs: shape, first-order, texture (GLCM, GLDM, GLRLM, GLSZM, NGTDM), PyRadiomics, Python (https://pyradiomics.readthedocs.io/en/2.1.2/, accessed on 23 May 2026).	reproducibility analysis + statistical tests + LASSO	SVM, kNN, LightGBM, LR	clinical model	0.747 (0.5660–0.9274)
				radiomics: LR	0.887 (0.7782–0.995)
				nomogram	0.900 (0.7874–1.0000)
Wu et al. [36]	1781 RFs: first order, LoG, wavelet, square, logarithm, squareRoot, exponential, gradient filtered, PyRadiomics (version 3.0.1), Python 3.7.6	mRMR	NB, Ensemble: Boosting + Bagging + RF, DT, LR, SVM: Linear + Polynomial + Gaussian, NN: 2 + 3 Layers, RFGB	radiomics: EP	0.921 (0.879–0.963)
Uhlig et al. [37]	127 RFs: first-order, 3D shape, 2D shape, GLCM, GLSZM, GLRLM, NGTDM, GLDM, 3D Slicer	no feature selection; RFE; PCA	XGBoost	radiomics	0.75
Yu et al. [38]	14,248 RFs: histogram, GLCM, GLSZM, GLRLM, NGTDM, GLCM	reproducibility analysis + statistical tests + LASSO	LR	clinical model	0.784 (0.740–0.828)
				radiomics	0.929
				nomogram	0.954 (0.933–0.975)
Yang et al. [39]	200 RFs (morphological + texture), PyRadiomics, Python, version 3.7 (https://www.python.Org, accessed on 23 May 2026) + 520 combined features	LASSO	LR, SVM, RF, XGBoost	radiomics: XGBoost + both CT phases	0.88 (0.82–0.95)
Maddalo et al. [40]	108 RFs: first-order, shape, GLCM, GLRLM, GLSZM, NGTDM, GLDM, SlicerRadiomics^®^	redundant elimination scaling + centering, balancing with RWO, reproducibility analysis	kNN	radiomics	0.79 ± 0.04
Garnier et al. [41]	>200 RFs: shape, intensity, texture	dimensionality reduction	Logit-LASSO, rpart, SVMLinear, RF, C5.0 Tree, wC5Tree	radiomics: C5.0Tree	0.736
Zhou et al. [42]	first-order, shape, GLCM, GLSZM, GLRLM, NGTDM, GLDM, Python (version 3.6.5), PyRadiomics	reproducibility analysis, CatBoost DT	CatBoost DT	radiomics, 3D + all CT phases	0.81
Feng et al. [43]	479 RFs: shape, first-order, texture, LoG	reproducibility analysis + statistical tests + LASSO	LR, DT	clinical model	0.814 (0.690–0.938)
				radiomics	0.954 (0.902–1.000)
				nomogram	0.968 (0.928–1.000)
Wentland et al. [44]	first-order, GLCM, GLSZM, GLRLM, PyRadiomics	RF	RF	radiomics signature: wavelet transform, RF	0.80
Nassiri et al. [45]	2D + 3D shape, texture features	reproducibility analysis	RF, real AdaBoost	clinical model	0.62 90.54–0.700
				radiomics	0.83 (0.77–0.88)
				nomogram	0.84 (0.79–0.90)
Yap et al. [46]	33 shape, 760 texture features	RF	RF, AdaBoost	radiomics: shape features, independent of CT phase, RF	0.68 (0.62–0.74)
Erdim et al. [47]	271 RFs/phase: histogram, gradient, run-length matrix, co-occurrence matrix, autoregressive, Haar wavelet, MaZda (version 4.6, P. M. Szczypinski, Institute of Electronics, Technical University of Lodz)	reproducibilty + Waikato Environment for Knowledge Analysis toolkit + correlation analysis	kNN, NN, LR, J48 DT, SVM, NB, LWL, RF	radiomics: CMP + RF	0.916
Uhlig et al. [48]	first-order, 3D shape, 2D shape, GLCM, GLSZM, GLRLM, NGTDM, GLDM, PyRadiomics	no feature selection; RFE; PCA	RF, RF ranger, XGBoost, boosted classification trees (C5.0), glmnet, SVM, kNN, NN	radiomics: XGBoost + no feature selection + SMOTE	0.72
Schieda et al. [49]	25 2D texture features: histogram, GLCM, RLM MaZda^®^, version 4.6 (P.M. Szczypiński, Institute of Electronics, Technical University of Lodz, Poland)	XGBoost	XGBoost	radiomics: all CT phases	0.73
Uhlig et al. [50]	120 RFs: first-order, 3D shape, 2D shape, GLCM, GLSZM, GLRLM, NGTDM, GLDM	RFE	XGBoost, RF, NN, SVM, kNN	radiomics: RF	**0.83**
Sun et al. [51]	first-order, shape, GLSZM, GLRLM, GLCM, CMP, PyRadiomics	reproducibility analysis + statistical testes + RFE-SVM	RFE-SVM	radiomics: combined model	**0.94 (0.89–0.96)**
Kunapuli et al. [52]	204 texture features: histogram, 2D/3D GLCM, 2D/3D GLDM, 2D FFT	RFE	RFGB	radiomics: RFGB	**0.83**

RFs: radiomics features; GLCM: Gray-Level Co-occurrence Matrix; GLRLM: Gray-Level Run-Length Matrix; GLSZM: Gray-Level Size Zone Matrix; NGTDM: Neighboring Gray Tone Difference Matrix; GLDM: Gray-Level Dependence Matrix; SVM: Support Vector Machine; RF: Random Forest; XGBoost: Extreme Gradient Boosting; LASSO: Least Absolute Shrinkage and Selection Operator; kNN: k-Nearest Neighbors; LightGBM: Light Gradient Boosting Machine; LR: Logistic Regression; LoG: Laplacian of Gaussian; mRMR: Minimum Redundancy Maximum Relevance; NB: Naive Bayes; DT: Decision Tree; RFGB: Random Forest-based Gradient Boosting; EP: excretory phase; RFE: Recursive Feature Elimination; PCA: Principal Component Analysis; RWO: Random Walk Oversampling; Logit-LASSO: Logistic Regression with LASSO regularization; rpart: Recursive Partitioning and Regression Trees; CatBoost: Categorical Boosting; AdaBoost: Adaptive Boosting; LWL: Locally Weighted Learning; SMOTE: Synthetic Minority Over-sampling Technique; AUC: Area Under the Curve; SD: standard deviation; CI: confidence interval.

**Table 7:** Segmentation strategies used to differentiate clear-cell from non-clear renal cell carcinoma.

Study	Segmentation Method
Yang et al. [39]	manual delineation, 3 radiologists + automated kidney and tumor segmentation, 3D-Unet
Sun et al. [51]	whole tumor, 3D semi-automated segmentation, 2 radiologists, Python (version 3.6.1, Python Software Foundation)
Cheng et al. [53]	whole tumor, manual 3D segmentation, 3 radiologists, 3D Slicer
Budai et al. [54]	whole tumor, not tumor margins, manual 3D segmentation, 2 radiologists, 3D Slicer, v.4.10.2
Gao et al. [55]	whole tumor, 1–2 mm from tumor margins, manual 3D segmentation, ITK-SNAP (version 3.8, www.itksnap.org, accessed on 23 May 2026)
Wu et al. [56]	automated segmentation, 3D-Unet
Zhang et al. [57]	whole tumor, 2 mm from tumor margins, manual 3D segmentation, 2 radiologists, ITK-SNAP (http://www.itk-snap.org, accessed on 23 May 2026)
Wang et al. [58]	whole tumor, 0–1 mm from tumor margins, manual 3D segmentation, 2 observers, ITK-SNAP
Chen et al. [59]	manual 3D segmentation, 2 mm from tumor margins, 2 radiologists, ITK-SNAP (www.itk-snap.org, accessed on 23 May 2026)
Li et al. [60]	whole tumor, manual 3D segmentation, ITK-SNAP
Kocak et al. [61]	largest tumor slice, 1–2 mm from tumor margins, manual 2D segmentation, MazDa (version 4.6, P. M. Szczypiński, Institute of Electronics, Technical University of Lodz)

**Table 8:** Radiomics pipeline for the differentiation of clear-cell from non-clear-cell renal cell carcinoma, including high-throughput radiomics features, optimal feature selection methods, machine learning algorithms, and final classification results.

Study	Extracted Features	Feature Selection	Model Training	Classification Results	AUC (±SD OR 95% CI)
Yang et al. [39]	200 RFs (morphological + texture), PyRadiomics, Python, version 3.7 (https://www.python.Org, accessed on 23 May 2026) + 520 combined features	LASSO	LR, SVM, RF, XGB	radiomics: XGBoost + both CT phases	0.90 (0.85–0.94)
Sun et al. [51]	RFs: first-order, shape, GLSZM, GLRLM, GLCM, PyRadiomics, CMP	reproducibility analysis + statistical tests + RFE-SVM	RFE-SVM	radiomics: combined model	0.93 (0.89–0.95)
Cheng et al. [53]	1168 RFs: original (first-order, GLCM, GLDM, GLRLM, GLSZM, NGDTM, shape 2D + 3D), LOG preprocessed + wavelet-transformed features, 3D Slicer	statistical tests + LASSO	LR, DT, RF, SVM, AdaBoost	radiomics: LR	0.929 (0.855–1.000)
				nomogram	0.949 (0.885–1.000)
Budai et al. [54]	321 RFs: first-order, shape, GLCM, GLRLM, GLSZM, GLDM, NGTDM	correlation, reproducibility analysis + LASSO or tuned ReliefF	SVM, RF	radiomics: SVM + CMP	0.834 (0.730–0.938)
Gao et al. [55]	1595 RFs/phase	reproducibility analysis + statistical tests + LASSO	LR	clinical model	0.717 (0.611–0.826)
				radiomics	0.821 (0.702–0.922)
				nomogram	0.831 (0.716–0.930)
Wu et al. [56]	520 RFs: texture, morphologic, statistical, PyRadiomics	n/a	XGBoost	radiomics	0.83 ± 0.1
Zhang et al. [57]	105 RFs/phase: first-order, 3D shape, texture (GLCM, GLSZM, GLRLM, GLDM, NGTDM), PyRadiomics	reproducibility analysis + LASSO	LR	clinical model	0.79
				radiomics: CMP/all CT phases	0.89
Wang et al. [58]	397 RFs: histogram, haralick, formfactor, GLSZM, GLCM, RLM (CMP)	reproducibility + correlation analysis + statistical tests	LR, RF, SVM	radiomics: RF	0.906
Chen et al. [59]	296 texture features: GLCM, GLRLM, GLSZM, NGTDM, GLDM, PyRadiomics	LASSO	LASSO	clinical model: CMP	0.823 (0.745–0.901)
				radiomics: same performance on CMP, NP, EP	0.887 (0.828–0.945)
				nomogram: same performance on CMP, NP, EP	0.900 (0.837–0.963)
Li et al. [60]	156 RFs: GLCM, GLRLM, GLSZM, NGTDM	reproducibility analysis + RF (Boruta) + mRMR	RF	radiomics: Boruta	0.949 (0.889–0.933)
				radiomics nomogram	0.951
Kocak et al. [61]	275 RFs/phase: histogram, gradient, GLCM, RLM, autoregressive model, Haar wavelet	reproducibility analysis + wrapper-based feature selection (ML classifiers)	MLP-ANN, SVM + SMOTE, adaptive boosting, bagging	radiomics: ANN + SMOTE, CMP	0.909

RFE-SVM: Recursive Feature Elimination-Support Vector Machine; RLM: Run-Length Matrix; ML: Machine Learning; MLP-ANN: Multilayer Perceptron Artificial Neural Networks; ANN: Artificial Neural Networks.

**Table 9:** Segmentation strategies used to differentiate fat-poor angiomyolipoma from renal cell carcinoma.

Study	Segmentation Method
Ma et al. [62]	whole tumor, 2–3 mm from tumor margins, manual 3D segmentation, 2 radiologists, ITK-SNAP (http://www.itksnap.org/; V 3.4.0, accessed on 23 May 2026)
Ma et al. [63]	whole tumor, 2–3 mm from tumor margins + peri-tumoral, 2 mm outside tumor margins: perirenal + perifat, manual 3D segmentation, 2 radiologists, mostly NP, ITK-SNAP (http://www.itksnap.org/, V3.4.0)
Ma et al. [64]	whole tumor, 2–3 mm from tumor margins, manual 3D segmentation, ITK-SNAP, version 3.4.0 (http://www.itksnap.org/, accessed on 23 May 2026)
Nie et al. [65]	whole tumor, manual 3D segmentation, 2 radiologists, ITK-SNAP (version 3.8, www.itksnap.org, accessed on 23 May 2026)
Yang et al. [66]	largest tumor slice, manual 2D segmentation, 2 radiologists, ITK-SNAP (http://www.itksnap.org, accessed on 23 May 2026)
Cui et al. [67]	whole tumor, 3 mm from tumor margins, manual 3D segmentation, 2 radiologists, ITK-SNAP (version 3.6.0, www.itksnap.Org, accessed on 23 May 2026)
Feng et al. [68]	largest tumor slice, 2–3 mm from tumor margins, manual 2D segmentation, 2 radiologists, DT kinetics
Lee et al. [69]	central tumor slice, manual 2D segmentation

**Table 10:** Radiomics pipeline for the differentiation of fat-poor angiomyolipoma from renal cell carcinoma, including high-throughput radiomics features, optimal feature selection methods, machine learning algorithms, and final classification results.

Study	Extracted Features	Feature Selection	Model Training	Classification Results	AUC (±SD OR 95% CI)
Ma et al. [62]	396 RFs: histogram, texture, form factor, GLCM, RLM	reproducibility + univariate + correlation analysis + LASSO	LR	radiomics	0.923 (0.797–0.982)
				nomogram	0.968 (0.923–0.990)
Ma et al. [63]	396 RFs/phase: histogram, texture, form factor, GLCM, GLRLM, GLZSM	reproducibility + univariate +correlation analysis + LASSO	LR	radiomics: tumoral + perirenal model	0.89 (0.842–0.927)
Ma et al. [64]	396 RFs: histogram, texture, form factor, GLCM, RLM, AK software (Artificial Intelligence Kit V 3.0.0, GE Healthcare)	statistical tests + correlation analysis + LASSO	LR	clinical model	0.935 (0.860–0.9770
				radiomics	0.925 (0.824–1.000)
				nomogram	0.988 (0.935–1.000)
Nie et al. [65]	2818 RFs: intensity, shape, texture (GLCM, GLRLM, GLSZM), filter, wavelet, CMP + NP, RadCloud platform (Huiying Medical Technology Co., Ltd)	reproducibility analysis + statistical tests + LASSO	n/a	clinical model	0.878 (0.718–1.000)
				radiomics	0.846 (0.634–1.000)
				nomogram	0.949 (0.856–1.000)
Yang et al. [66]	103 RFs: shape, first-order, texture, PyRadiomics	28 feature selection methods	LR, SVM, NB, kNN, DT, bagging, RF, AdaBoosting	radiomics: SVM + t_score (UECT), SVM + relief (UECT + NP)	0.90
Cui et al. [67]	original features: first-order, shape, GLCM, GLSZM, GLRLM, NGTDM, GLDM + filtered: wavelet, LoG, square, squareroot, logarithm, exponential, PyRadiomics	SMOTE + Boruta package + SVM-RFECV	SVM-RFECV	clinical model: fpAML vs. RCC	0.67
				clinical model: fpAML vs. ccRCC	0.68
				radiomics: Dd fpAML vs. RCC	0.96
				radiomics: Dd fpAML vs. ccRCC	0.97
Feng et al. [68]	42 RFs: histogram, GLCM	reproducibility analysis + statistical tests + SVM-RFE	SVM-RFE + SMOTE	radiomics: SVM-RFE + SMOTE	0.955 (0.855–0.988)
Lee et al. [69]	71 RFs: histogram, texture, shape	statistical tests	RF	radiomics	0.774

RFECV: Recursive Feature Elimination with Cross-Validation.

**Table 11:** Segmentation strategies used to differentiate renal oncocytoma from renal cell carcinoma.

Study	Segmentation Method
Ye et al. [70]	whole tumor manual 3D segmentation, 2 radiologists, ITK-SNAP (http://www.itksnap.org, accessed on 23 May 2026) + peritumoral: 1mm, 2mm, 3mm beyond tumor margins, “scipy.ndimage”
Yang et al. [71]	whole tumor, 1mm from tumor margins, manual 3D segmentation, 2 radiologists, 3D Slicer (https://www.slicer.org/), accessed on 23 May 2026
Aymerich et al. [72]	whole tumor, manual 3D segmentation, 3 radiologists
Carlini et al. [73]	whole tumor + tumor’s zone of transition, manual 3D segmentation, multiple observers, D2P^TM^ (‘DICOM to PRINT’; 3D Systems Inc., Rock Hill, SC, USA)
Yu et al. [74]	whole tumor, manual 3D segmentation, 3 observers, ITK-SNAP version 4.11.0 (www.itk-snap.Org, accessed on 23 May 2026)
Alhussaini et al. [75]	whole tumor, 2mm from tumor margins, manual 3D segmentation, Python + whole tumor, 3D semi-automated segmentation, 2 observers
Li et al. [76]	whole tumor, manual 3D segmentation, 2 radiologists
Li et al. [77]	whole tumor, manual 3D segmentation, 2 observers, 3D Slicer (version 4.10.2, https://www.slicer.Org, accessed on 23 May 2026)
Jaggi et al. [78]	Radiomic Biopsy: a spherical sample, or a cluster of connected spherical samples, of a Volume of Interest, Fovia’s FAST (Fovia Inc., Palo Alto, CA, USA)
Li et al. [79]	whole tumor, manual 3D segmentation, 2 radiologists, RadCloud (Big Data Intelligent Analysis Cloud Platform, Huiying Medical Technology Co., Ltd., Beijing, China)
Yu et al. [80]	10 consecutive axial slices (tumor mid-portion), manual 3D segmentation

**Table 12:** Radiomics pipeline for the differentiation of renal oncocytoma and renal cell carcinoma, including high-throughput radiomics features, optimal feature selection methods, machine learning algorithms, and final classification results.

Study	Extracted Features	Feature Selection	Model Training	Classification Results	AUC (±SD OR 95% CI)
Ye et al. [70]	2260 tumoral + 6780 peritumoral RFs: shape, texture, first-order, LoG, GLCM, GLRLM, GLSZM, NGTDM, GLDM + exponential, gradient, square, wavelet transforms, PyRadiomics	reproducibility analysis + statistical tests + LASSO	SVM	radiomics	0.900 (0.792–1.00)
Yang et al. [71]	1278 RFs: first-order, shape, texture (GLCM, GLSZM, GLRLM, NGTDM, GLDM), wavelet, LοG, PyRadiomics	reproducibility + correlation analysis + LASSO	LR	radiomics	0.84 (0.69–0.99)
				nomogram	0.93 (0.83–1.00)
Aymerich et al. [72]	105 RFs: shape, first-order, GLCM, GLRLM, GLSZM, NGTDM, Quibim Precision 2.8 (Quibim S.L., Valencia, Spain)	reproducibility + correlation analysis + statistical tests	LR, RF, SVM	radiomics: LR	0.75 (0.55–1.00)
Carlini et al. [73]	2436 RFs: 2D, 3D, Laplacian, wavelet (CMP), PyRadiomics	genetic algorithm	DT	radiomics: ZOT features, feature selection + whole data set	0.87 ± 0.09
Yu et al. [74]	396 RFs/phase: histogram, texture, GLCM	reproducibility analysis + mRMR + LASSO	SVM	clinical model	0.630 (0.240–1.000)
				nomogram	0.950 (0.850–1.000)
Alhussaini et al. [75]	204 original: first-order, GLCM, GLRLM, GLSZM, NGTDM, GLDM, shape + 3.180 filtered: wavelet, LoG, square, logarithm, square-root, gradient exponential, LBP 2D/3D PyRadiomics Python version 3.6.1	LASSO	RF, SVM, LR, kNN, NB	signature: whole tumor volume + RF	1.00 ± 0.000
Li et al. [76]	851 RFs/phase: 107 original + 744 wavelet-filtered, 3D Slicer (Pyradiomics v. 2.2.0, accessed on 23 May 2026)	reproducibility analysis + statistical testes + LASSO	LR	clinical model	0.895 (0.796–0.993)
				radiomics	0.957 (0.904–1.000)
				nomogram	0.988 (0.966–1.000)
Li et al. [77]	2553 RFs	reproducibility analysis + statistical tests + LASSO	LR	clinical model	0.761 (0.609–0.913(
				radiomics	0.842 (0.684–0.999)
				nomogram	0.898 (0.791–1.000)
Jaggi et al. [78]	6.206 RFs: intensity, texture, quantitative image feature engine	reproducibility analysis + mRMR	RF + adaBoost	radiomics	0.71 ± 0.024
Li et al. [79]	1029 RFs/phase: intensity, shape, texture (GLCM, GLRLM, GLSZM), high-order (Laplacian, exponential, logarithmic, square, square root, wavelet), RadCloud (Big Data Intelligent Analysis Cloud Platform, Huiying Medical Technology Co., Ltd., Beijing, China)	LASSO + statistical tests	kNN, SVM, RF, LR, MLP	radiomics: SVM, CMP + NP	0.964 ± 0.054
Yu et al. [80]	43 texture features: histogram, GLCM, GLRL, GLG, Laws’ features, MATLAB-based (R2015b, Mathworks Inc., Natick, MA, USA)	SVM	SVM	radiomics: RO vs. RCC	0.917(0.856–0.978)
				radiomics: RO vs. chRCC	0.882 (0.764–1.000)

ZOT: zone of transition; LBP: local binary patterns; MLP: Multilayer Perception; GLG: Gray-Level Gradient.

## Data Availability

Data supporting this study are included within the article and/or Supplementary Materials.

## Supplementary Materials

The following supporting information can be downloaded at: https://www.mdpi.com/article/10.3390/cancers18111758/s1, Table S1. Description of CT imaging parameters utilized for the differential diagnosis of benign renal tumors and renal cell carcinoma. Table S2. Description of CT imaging parameters utilized for the differential diagnosis of clear-cell and non-clear-cell and renal cell carcinoma. Table S3. Description of CT imaging parameters utilized for the differential diagnosis of fat-poor angiomyolipoma and renal cell carcinoma. Table S4. Description of CT imaging parameters utilized for the differential diagnosis of renal oncocytoma and renal cell carcinoma. Table S5. Non-radiomics models used for the characterization of renal tumors.

## Abbreviations

RCC	renal cell carcinoma
ccRCC	clear cell renal cell carcinoma
pRCC	papillary renal cell carcinoma
chRCC	chromophobe renal cell carcinoma
fpAML	fat-poor angiomyolipoma
RO	renal oncocytoma
PN	partial nephrectomy
CECT	contrast-enhanced CT
ML	Machine Learning
VOI	Volume of Interest
AUC	Area Under the Curve
PRISMA	Preferred Reporting Items for Systematic Reviews and Meta-Analyses
kV	kilovolt
mA	milliampere
iv	intravenous
RF	radiomics feature
METRICS	METhodological RadiomICs Score
SD	standard deviation
IQR	interquartile range
M/F	male/female
TCIA	The Cancer Imaging Archive
CMP	corticomedullary phase
NP	nephrographic phase
UECT	unenhanced CT
EP	excretory phase
3D	three-dimensional
3D	two-dimensional
GLCM	Gray-Level Co-occurrence Matrix
GLRLM	Gray-Level Run Length Matrix
GLSZM	Gray-Level Size Zone Matrix
NGTDM	Neighboring Gray-Tone Difference Matrix
GLDM	Gray-Level Difference Matrix
LASSO	Least Absolute Shrinkage and Selection Operator
RF	Random Forest
XGBoost	extreme Gradient Boosting
RFE-SVM	Recursive Feature Elimination-Support Vector Machines
kNN	k-Nearest Neighbors
LR	Logistic Regression
DT	Decision Tree
LoG	Laplacian of Gaussian
KiTS19	Kidney Tumor Segmentation Challenge 2019
TCGA	The Cancer Genome Atlas
NN	Neural Networks
