# Supplementary material for: CT-Based Radiomics in the Characterization of Solid Renal Tumors: A Systematic Review
Source: Cancers (Basel). 2026 May 27;18(11):1758. doi: 10.3390/cancers18111758 (PMC13255990; doi:10.3390/cancers18111758)
Supplement: Supplementary file 1 [file cancers-18-01758-s001.zip › cancers-4275777-supplementary/SUP/cancers-4275777 Supplementary material.pdf]

Supplementary Material

Table S1. Description of CT parameters utilized for the differential diagnosis of benign renal tumors and renal cell carcinoma

Study	CT scanner	Slice thickness (mm)	kV	mAs	Type of iv cm (mgI/ml)	Amount of cm	Rate of injection (ml/s)	Phases	Timing of phases (s)
**Bang et al [34]**	n/a	n/a	n/a	n/a	n/a	n/a	n/a	UECT, CMP, NP, EP	20-30, 60-70, > 180
**Qian et al [35]**	MDCT	1.5	120	150-200	Iohexol 350	1.5 ml/kg	3.5	CMP, NP, EP	20-25, 70-90, 180
**Wu et al [36]**	various	3-7	100-140	200-720	n/a	n/a	n/a	CMP, NP, EP	25-35 70-90 160-180
**Uhlig et al [37]**	various	n/a	n/a	n/a	Iopromide 370	1.1 ml/kg	3	CMP, NP	(aorta, > 120 HU) +10 +40
**Yu et al [38]**	various	n/a	120	200-350	Iohexol 350	1.5 ml/kg	3	UECT, CMP, NP	30, 60-70
**Yang et al [39]**	various	0.5-5	120	200-360	n/a	n/a	n/a	CMP, NP	n/a
**Maddalo et al [40]**	16- 64-MDCT, DECT	1.5-2.5	100-130	n/a	Iomeron 300	90-130 ml	3.0-4.0	NP	60
**Garnier et al [41]**	64-MDCT	n/a	120	500	350-400	80 ml	3.5-4.0	CMP, NP, EP	(aorta, 100 HU) +10 +100 600-900
**Zhou et al [42]**	16- 64-MDCT, dual source CT	1 or 3	120	250-350	Iopamidol	70-100 ml	3.0	UECT, CMP, NP	30, 90

**Feng et al [43]**	256-MDCT	3	120	n/a	Ioversol	80 ml	3.3	CMP, NP, EP	30, 65, 180
**Wentland et al [44]**	MDCT, various	0.625-5	100-140	n/a	Iohexol 300	n/a	n/a	n/a	n/a
**Nassiri et al [45]**	64-MDCT (54%)	0.5	120	n/a	Iopamidol 350	n/a	4.0-5.0	UECT, CMP, NP, EP	n/a
**Yap et al [46]**	64 MDCT (42%)	0.5	120	n/a	Iopamidol 350	100–150 mL	4.5	UECT, CMP, NP, EP	30, 90, 300-420
**Erdim et al [47]**	128- 64-MDCT	1-3	120	100–500	n/a	1-2 ml/kg	n/a	UECT, CMP	n/a
**Uhlig et al [48]**	various	2 (1-5)	n/a	n/a	n/a	n/a	n/a	NP	n/a
**Sheida et al [49]**	16- 64- 320-MDCT	2.5	120	150-450	Iopamidol	105 ml	3,5	UECT, CMP, NP	30-40 110-120
**Uhlig et al [50]**	various	2.5	n/a	n/a	n/a	n/a	n/a	NP	n/a
**Sun et al [51]**	128-MDCT	n/a	120	180	Iopromide 300	90-120 ml	3.0	UECT, CMP, NP	(aorta, 100 HU) + 10 + 40
**Kunapuli et al [52]**	64-MDCT	0.5	120	n/a	n/a	n/a	n/a	UECT, CMP, NP, EP	30, 90, 300-420

MDCT: multidetector CT; DECT: dual-energy CT; n/a: non-applicable; kV: kilovolt; mAs: milliamperes; mgI/ml: milligrams of iodine per milliliter; iv: intravenous; ml: milliliters; kg: kilogram; cm: contrast medium; UECT: unenhanced CT; CMP: corticomedullary phase; NP: nephrographic phase; EP: excretory phase; HU: Hounsfield Unit

Table S2. Description of CT parameters utilized for the differential diagnosis of clear cell and non-clear cell and renal cell carcinoma

Study	CT scanner	Slice thickness (mm)	kV	mAs	Type of iv cm (mgI/ml)	Amount of cm	Rate of injection (ml/s)	Phases	Timing of phases (s)
**Yang et al [39]**	various	0.5-5	120	200-360	n/a	n/a	n/a	CMP, NP	n/a
**Sun et al [51]**	128-MDCT	n/a	120	180	Iopromide 300	90-120 ml	3.0	UECT, CMP, NP	(aorta, 100 HU) + 10 + 40
**Cheng et al [53]**	various	n/a	n/a	n/a	n/a	n/a	2.5-3.5	CMP	25-30
**Budai et al [54]**	16 64-MDCT	1.25	100-140	105-977	350–370 mg/ml	0.5 gr/kg	1.5–3.5 ml/s	UECT, CMP, EP	30–45, 300–480
**Gao et al [55]**	various	5.1	120	180-450	Iohexol 320	1.5 ml/kg	3.0	UECT, CMP, NP, EP	30, 80, 180
**Wu et al [56]**	various	n/a	n/a	n/a	n/a	n/a	n/a	CMP, NP	n/a
**Zhang et al [57]**	64- 256-MDCT	5	120	150-320	Iohexol or Iopromide	2 ml/kg	2,5	UECT, CMP, NP, EP	30-35, 50-60, 190-200
**Wang et al [58]**	64-MDCT	5	100–120	134–409	Iodophor alcohol	0.9-1.01 ml/kg	3.0-3.5	CMP, NP, EP	30-35, 50-60, 180
**Chen et al [59]**	64- 256-MDCT	5	120	150-320	Iohexol, Iopromide	2 ml/kg	2.5	UECT, CMP, NP, EP	30-35, 60-70, 190-200
**Li et al [60]**	various	5	120	110-400	Iopamidol or Iopromide	400-500 ml	n/a	UECT, CMP, NP	30, 65-75
**Kocak et al [61]**	64- 128-MDCT	1-2	120	100-500	n/a	2 ml/kg	n/a	UECT, CMP	60

Table S3. Description of CT parameters utilized for the differential diagnosis of fat-poor angiomyolipoma and renal cell carcinoma

Study	CT scanner	Slice thickness (mm)	kV	mAs	Type of iv cm (mgI/ml)	Amount of cm	Rate of injection (ml/s)	Phases	Timing of phases (s)
**Ma et al [62]**	64- 128-MDCT	5	120	200	Iopromide 370	90–100 ml	3.0	UECT, CMP, NP	(aorta, 100 HU) +15 +30
**Ma et al [63]**	64- 128-MDCT	5	120	200	Iopromide	90-100 ml	3.0	UECT, CMP, NP	(aorta, 100 HU) +15 +30
**Ma et al [64]**	64- 128-MDCT	5	120	200	Iopromide, 370	90-110 ml	3.0	UECT, CMP, NP	(aorta, 100 HU) +15 +30
**Nie et al [65]**	64-MDCT	5	120	200-400	Iopromide 370	90-100 ml	2.5	UECT, CMP, NP, EP	30, 90, 300-420
**Yang et al [66]**	various	5	120-140	n/a	n/a	n/a	n/a	UECT, CMP, NP, EP	n/a
**Cui et al [67]**	64-MDCT	1 or 3	120	200	Iopamidol or Iohexol	70-100 ml	3	UECT, CMP, NP	30, 90
**Feng et al [68]**	64-MDCT	5	120	200	Iohexol 350	90 mL	3.0	UECT, CMP, NP	30 ,90
**Lee et al [69]**	various	1-3	n/a	n/a	Iobitridol	2 ml/kg	3.0	NP	100-120

Table S4. Description of CT parameters utilized for the differential diagnosis of renal oncocytoma and renal cell carcinoma

Study	CT scanner	Slice thickness (mm)	kV	mAs	Type of iv cm (mgI/ml)	Amount of cm	Rate of injection (ml/s)	Phases	Timing of phases (s)
Ye et al [70]	various	1-5	100-140	100-250	Iopromide 370, Ioversol 350, Iohexol 350	100-120	3.0-4.0	CMP, NP	aortic triggering, 60-70
Yang et al [71]	256 iCT, 320-detector	5	120	220-250	Iopromide 300	1.5 ml/kg	3	UECT, CMP, NP	30-35 60-70
Aymerich et al [72]	various	n/a	n/a	n/a	n/a	n/a	n/a	NP	90
Carlini et al [73]	6- 16-64-MDCT	2.5-5	120-140	120	Iomeprol 300/350 Iomeron	120-140 ml	3	UECT, CMP, NP, EP	25-30, 80-100, 300-600
Yu et al [74]	256-MDCT	5	120	100	300	1.5ml/kg	n/a	CMP, NP	25-30, 60-70
Alhussaini et al [75]	helical CT	1.25	120	100-560	Iohexol 300	80-100 ml	3.0	NP	100-120
Li et al [76]	n/a	2.5	120	200-400	Iopromide 370	80-100 ml	3.0	CMP, NP, EP	35-40, 90, 420-540
Li et al [77]	64-MDCT	2.5	120	n/a	Iopromide 370	80-100 ml	3.0	CMP, NP, EP	35-40, 90, 420-540
Jaggi et al [78]	various	5	n/a	n/a	Iopamidol 370	150 ml	4	NP	90
Li et al [79]	64-MDCT	5-8	120	n/a	n/a	n/a	3.0	CMP, NP, EP	30-200
Yu et al [80]	64-MDCT	1.25	120	200–650	Ioversol 350 and Iopamidol 370	100	3.0	NP	70

Table S5. Non-radiomics models used for the characterization of renal tumors

Study	Non-radiomics model
**Dd benign renal tumors vs RCC**	
**Qian et al [35]**	sex, age, history of smoking, drinking, hypertension, diabetes, tumor shape
**Garnier et al [41]**	sex, age, BW, ECOG score, GFR, symptoms, clinical TNM + infiltration, contour, homogeneity, calcifications, fat, hemorrhage, necrosis, venous extension, multifocality, bilaterality, enhancement
**Zhou et al [42]**	shape, heterogeneity, septa, extrarenal extension, calcification, hemorrhage, necrosis, internal arteries, pseudocapsule
**Feng et al [43]**	sex, age, size
**Nassiri et al [45]**	age, gender, smoking, comorbid conditions, symptoms, family history of RCC, baseline Cre, chronic kidney disease, laterality
**Sun et al [51]**	age, sex, laterality, location, size, shape, margin, psedocapsule, extrarenal extension, calcification, hemorrhage, necrosis, heterogeneity, septa, internal arteries, enhancement
**Dd ccRCC vs non-ccRCC**	
**Sun et al [51]**	age, sex, laterality, location, size, shape, margin, psedocapsule, extrarenal extension, calcification, hemorrhage, necrosis, heterogeneity, septa, internal arteries, enhancement
**Cheng et al [53]**	age, gender, intratumoral vessels, enhancement, renal vein invasion, size
**Gao et al [55]**	age, sex, size, shape, laterality, margins, calcification, necrosis, renal vein invasion, lymph node metastasis, enhancement
**Zhang et al [57]**	age, sex, symptoms, laterality, location, cystic components, calcifications, TNM, maximum tumor diameter, CT attenuation of solid portion
**Chen et al [59]**	tumor enhancement
**Li et al [60]**	age, sex
**Dd fpAML vs RCC**	
**Ma et al [62]**	laterality, pseudocapsule, cyst, calcification, enhancement pattern, CT density
**Ma et al [64]**	location, size, growth patern, angular interface, cyst degeneration, calcification, pseudocapsule, enhancement pattern, gender, CT density
**Nie et al [65]**	age, gender, height, weight, BMI + size, shape, location, angular interface, dysmorphic vessels, fast in, fast out enhancement
**Cui et al [67]**	CT density, exophytic growth, homogeneity of enhancement

	Dd RO vs RCC
**Yang et al [71]**	age, laterality, size
**Yu et al [74]**	age, enhancement
**Li et al [76]**	gender, age, laterality, location, segmental enhancement inversion, necrosis, cystic components, hemorrhage, calcification, fat, perirenal fascia thickening
**Li et al [77]**	gender, age, location, central scar, cystic components, perirenal fascia thickening

Dd: differential diagnosis; RCC: renal cell carcinoma; ccRCC: clear cell renal cell carcinoma; non-ccRCC: non- clear cell renal cell carcinoma; fpAML: fat-poor angiomyolipoma; RO: renal oncocytoma; BW: body weight; ECOG: Eastern Cooperative Oncology Group; GFR: Glomerular Filtration Rate; BMI: Body Mass Index; TNM: Tumor Node Metastasis; Cre: creatinine
